# The WheelCams on the IDEFIX rover

**DOI:** 10.1186/s40645-025-00725-3

**Published:** 2025-07-14

**Authors:** Naomi Murdoch, Valérian Lalucaa, Cecily Sunday, Simon Tardivel, Jean Bertrand, Nicolas Théret, Damien Vivet, Alice Amsili, Colas Robin, Panos Delton, Alexia Duchene, Quentin Douaglin, Antoine Maillard, Cedric Virmontois, Pierre Vernazza, Laurent Jorda, Olivier Groussin, Hideaki Miyamoto, Jean-Baptiste Vincent, Jessica Flahaut, Jens Biele, Olivier Barnouin, Christine Hartzell, Fabien Buse, Stefan Barthelmes, Stephan Ulamec, Patrick Michel, Julien Baroukh

**Affiliations:** 1 ISAE-SUPAERO, Université de Toulouse, 10 Avenue Marc Pélegrin, 31055 Toulouse, France; 2 CNES, 18 Avenue Edouard Belin, 31401 Toulouse, France; 3 University of Maryland, College Park, MD USA; 4 Thales Services Numériques, 3 Av. de l’Europe, 31400 Toulouse, France; 5 Aix Marseille Université, CNRS, CNES, LAM, 38 rue Frederic Joliot Curie, 13013 Marseille, France; 6 University of Tokyo, Tokyo, Japan; 7 German Aerospace Center, DLR, Berlin, Germany; 8 Université de Lorraine, CNRS, CRPG, Nancy, France; 9 German Aerospace Center, DLR, 51147 Cologne, Germany; 10 APL, Los Angeles, CA USA; 11 Université Côte d’Azur, Observatoire de la Côte d’Azur, CNRS, Laboratoire Lagrange, 06304 Nice, France

**Keywords:** Phobos, MMX, Camera, In situ imaging, Regolith, Locomotion, Terramechanics, Geotechnics, Morphology, Space weathering

## Abstract

IDEFIX, the Martian Moons eXploration (MMX) mission Phobos rover, will be the first of its kind to attempt wheeled-locomotion on a low-gravity surface. The IDEFIX WheelCams, two cameras placed on the underside of the rover looking at the rover wheels, provide a unique opportunity to study the surface properties of Phobos, regolith behaviour on small-bodies and rover mobility in low-gravity. The information gained about Phobos’ surface will be of high importance to the landing and sampling operations of the main MMX spacecraft, in addition to being valuable for understanding the surface processes and geological history of Phobos. Here we introduce the WheelCam science objectives, the instrument and the characterisation activities. We also discuss the on-going preparations linked to the analysis and interpretation of the WheelCam images on the surface of Phobos.

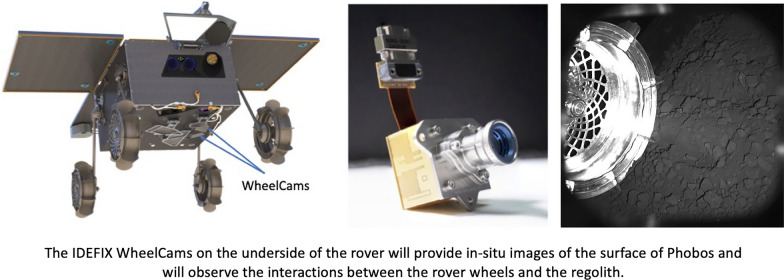

## Introduction

The JAXA Martian Moons eXploration (MMX) mission (Kuramoto et al. 2022) will deploy the French–German IDEFIX rover to the surface of Phobos (Michel et al. 2022). The IDEFIX rover will attempt wheeled-locomotion on a small body surface, in a very low-gravity environment, for the first time, thus providing a unique opportunity to study the surface properties of Phobos and the behaviour of regolith on small-bodies.

The physical properties of Phobos’ surface are closely connected to the moon’s history and origin. Understanding these properties is crucial for spacecraft–surface interactions, such as the landing of the main MMX spacecraft and surface sampling. Since remote observations can sometimes yield differing interpretations of surface and internal characteristics, direct interaction with the surface material is the most reliable way to investigate its mechanical and physical properties.

Space agencies have visited asteroids and small bodies in the past, but have only interacted with their surfaces using landers, hopping rovers, and touch-and-go maneuvers (e.g. the Philae lander on the Rosetta mission, the Mascot and Minerva rovers on the Hayabusa2 mission, and the sampling mechanisms on the Hayabusa, Hayabusa2 missions and OSIRIS-REx missions).

**Fig. 1:**
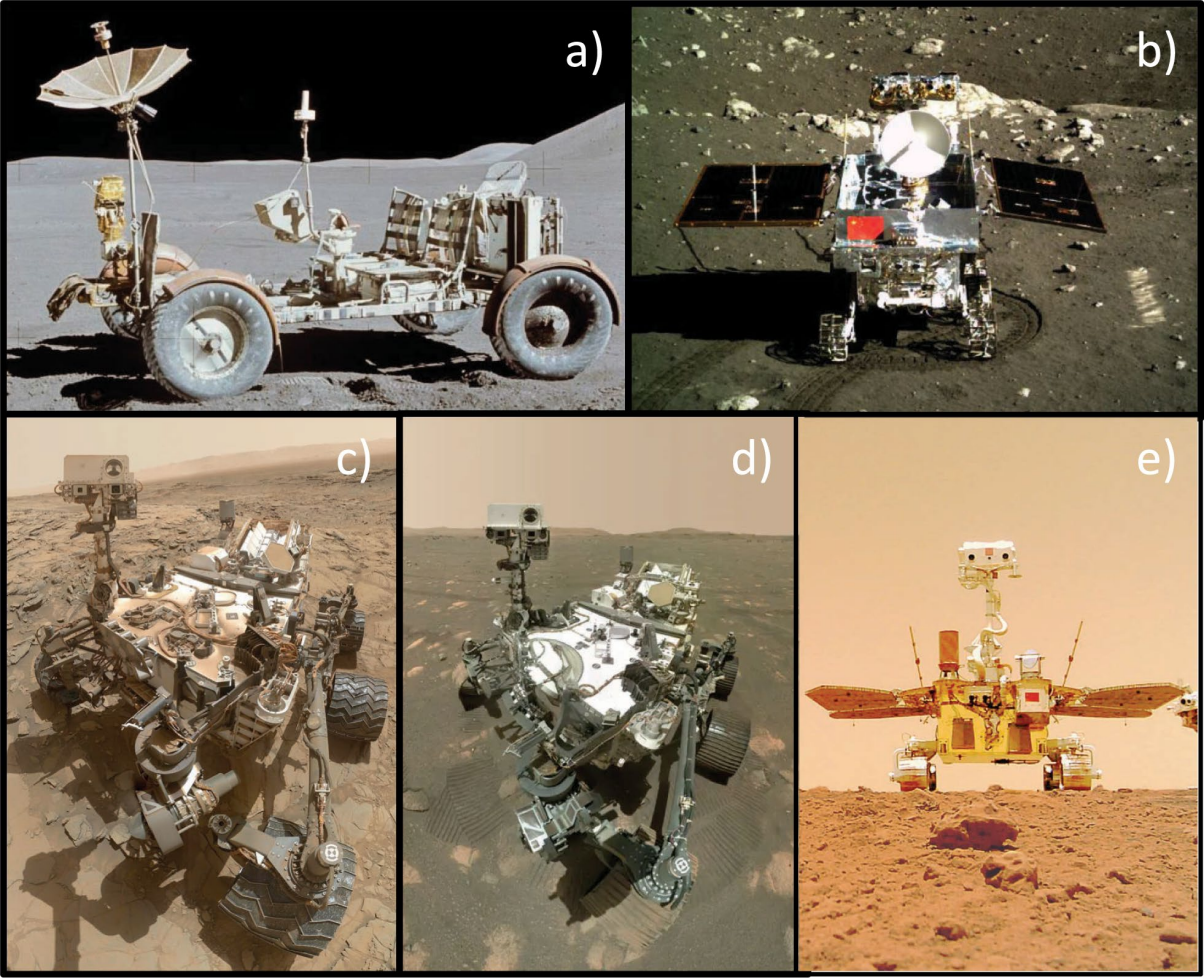
Examples of wheeled rovers on the surface of the Moon and Mars. **a** The Lunar Roving Vehicle (NASA) was operated during the Apollo program (Asnani et al. 2009). **b** The Yutu-1 rover (CNSA) was developed for the Chang’E-3 lunar mission (Li et al. 2015). The **c** Curiosity (Grotzinger et al. 2012; Vasavada 2022) and **d** Perseverance (Farley et al. 2020) Mars rovers developed by NASA. **e** The Zhurong rover developed by CNSA for the Tianwen-1 Mars mission (Mallapaty 2021; Ding et al. 2021b)

Wheeled rovers have proven to be useful tools for identifying the surface material properties of the Moon and Mars (Fig. 1; Arvidson et al. 2003; Sullivan et al. 2011; Gao et al. 2016; Ding et al. 2021b). However, as the effective gravitational acceleration on Phobos (including Phobos’ gravity but also centrifugal acceleration and Mars’ tides) is ~600$\times$ smaller than that of Mars and $\sim$300$\times$ smaller than that of the Moon, the lessons learned about wheeled locomotion from previous rover missions may be of limited use to the IDEFIX mission. Still, experiments, simulations, and scaled models can be used to prepare for the rover surface operations, and to ensure that the observed regolith interactions are correctly interpreted, taking into account the low-gravity environment.

The MMX mission is planned for launch in 2026, with an expected arrival in orbit around Mars in July 2027, followed by orbit insertion around Phobos in September 2027 (Kawakatsu 2025). After a period of Phobos observations, the IDEFIX rover will be deployed late 2028/early 2029 to the surface of Phobos. After being deployed from the main MMX spacecraft, IDEFIX will first go through the Separation, Landing, Uprighting and Deployment (SLUD) phase. This is a fully autonomous sequence during which wheel & leg actuation will be used to place the rover into the correct - upright - orientation. An initial mobility and locomotion verification phase will follow during which the first Phobos drives will occur. This phase will allow the regolith-wheel interactions to be tested providing the first information about driving in extremely low gravity conditions. The IDEFIX rover will then operate on the surface of Phobos for approximately 100 days. In that time the rover is expected to cover a distance of 10 to 100 m. For more information about the IDEFIX operations see Ulamec et al. (2024). The Phobos sampling (by the main MMX spacecraft) will occur in spring/summer of 2029. MMX will then leave Phobos and perform multiple flybys of Deimos in spring/summer of 2030 before ejecting the return module. The samples are expected to arrive back on Earth in June 2031 (Kuramoto et al. 2022).

**Fig. 2:**
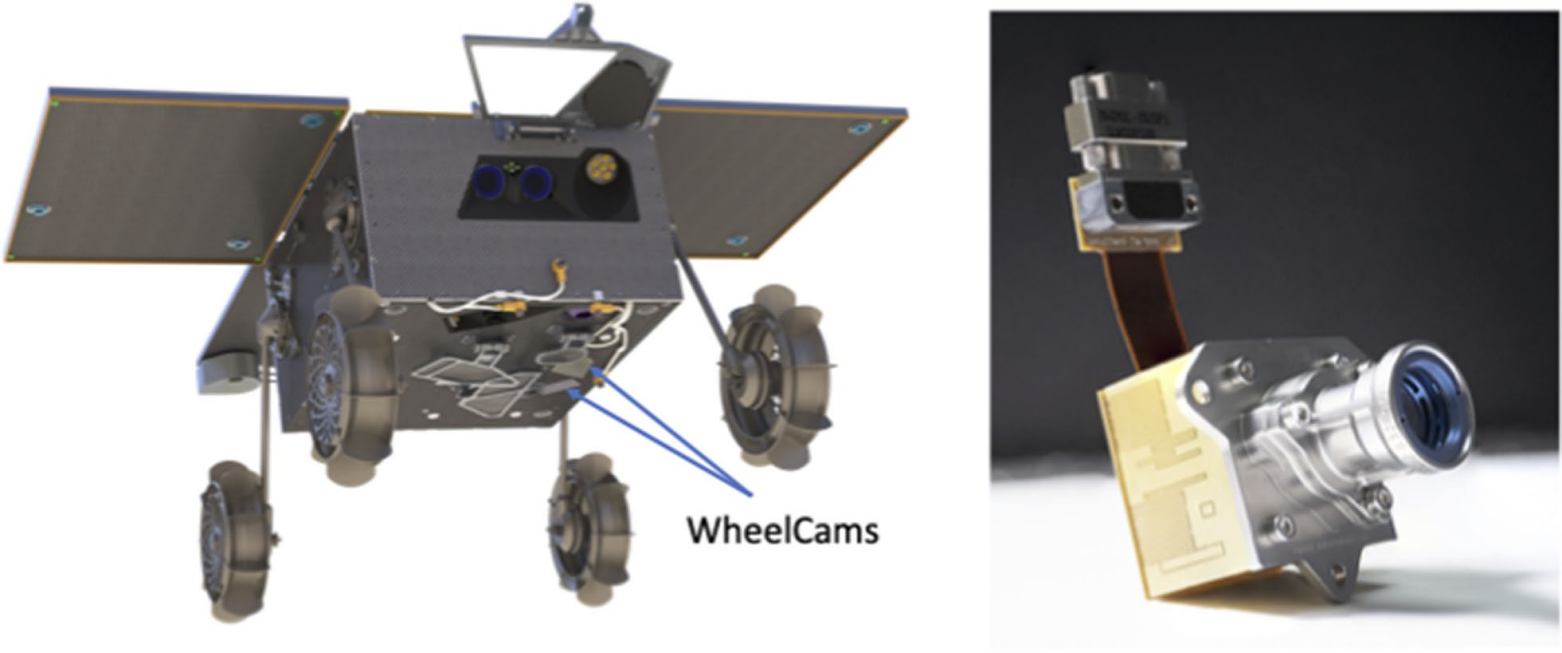
The WheelCams on IDEFIX. Left: The MMX IDEFIX rover showing the location of the two WheelCams. Right: The flight model of a WheelCam. Image credit: CNES

The IDEFIX locomotion subsystem, which will be used for unfolding, standing up, driving, aligning and lowering the rover has been specifically designed for Phobos (Barthelmes et al. 2022). The shape of the IDEFIX rover wheels (Fig. 2, left) was designed using an optimisation algorithm combined with Discrete Element Method simulations (Stubbig and Lichtenheldt 2021). The WheelCams consist of a set of two cameras, each with a set of co-located Light Emitting Diodes (LEDs), placed on the underside of the IDEFIX rover (Fig. 2). The front WheelCam observes the left front wheel, and the rear WheelCam observes the front of the left back wheel and also the trench made by the left front wheel. The WheelCams will provide in-situ images of the surface of Phobos and will allow us to examine the mechanical and dynamic properties of Phobos’ regolith by observing the surface and the interactions between the rover wheels and the regolith.

In this paper we describe the science objectives of the IDEFIX WheelCam instrument (Sect. 2), before presenting the technical details of the instrument and the planned instrument operations (Sect. 3), and the characterisation tests (Sect. 4). We end by describing the on-going activities in preparation for the IDEFIX rover operations and WheelCam analyses on the surface of Phobos (Sect. 5).

## WheelCam scientific objectives

The main MMX mission objectives are to understand the origin of Phobos and Deimos, in addition to the surface evolution and processes (Kuramoto et al. 2022). The IDEFIX rover measurements will be used to support the interpretation of data obtained by instruments onboard the main MMX spacecraft, and to minimize the risks involved in the MMX spacecraft sampling operations.

The IDEFIX science objectives are defined as follows (Michel et al. 2022):

1. Determine the composition and formation conditions of the surface of Phobos.
2. Determine the origin of the blue and red materials on Phobos.
3. Determine the internal structure/sub-surface properties and constrain the global physical properties.
4. Correlate mineral/rock types and relative abundances of Phobos and Mars materials.
5. Correlate the mineralogy derived from measurements on Phobos with that derived from returned samples.
6. Characterisation of physical properties and dynamics of regolith.
7. Characterisation of surface alteration.
8. Characterisation of Phobos’ grooves.

The specific IDEFIX WheelCam science objectives are:

(i) Determine the physical properties of the regolith particles.
(ii) Determine the bulk mechanical properties of the regolith.
(iii) Determine the dynamical behaviour of the regolith.
(iv) Observe possible layering in the shallow sub-surface.
(v) Constrain the mineralogical composition of the surface material.
(vi) Assess space weathering.
(vii) Determine regolith geological classes.
(viii) Constrain the absolute local gravitational acceleration.

These objectives and the corresponding analysis techniques are given in Table 1. Additional information is provided in the following sub-sections.

### Physical properties of regolith particles

#### Particle size and shape distribution

**Fig. 3:**
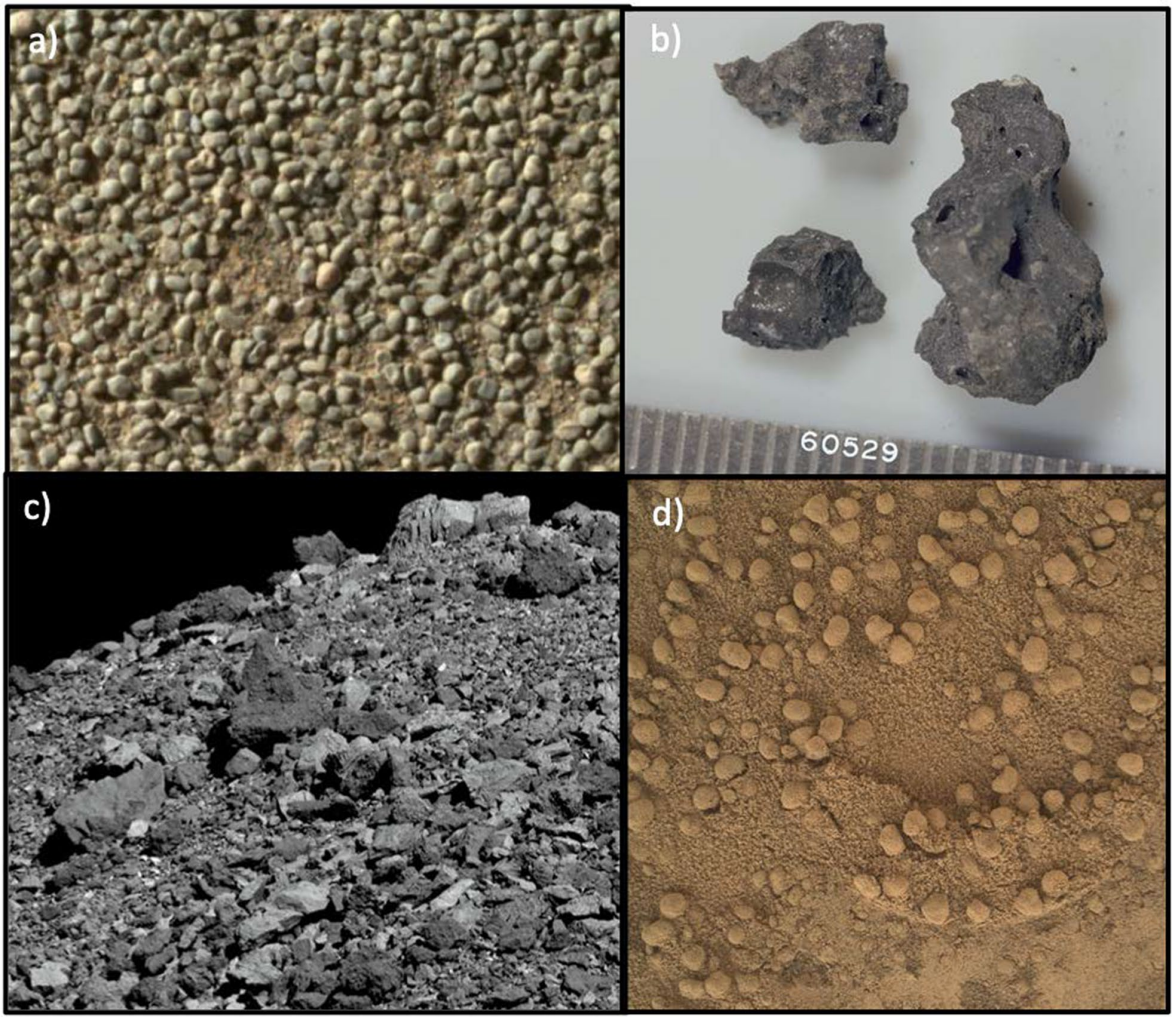
Varied particle morphologies on different planetary surfaces. **a** Image of rounded coarse grains present on the flank of a small ripple as oberved by Wide Angle Topographic Sensor for Operations and eNgineering (WATSON) on the NASA Mars 2020 Perseverance rover on sol 106 (image from Vaughan et al. 2023). **b** Apollo 16 Lunar Sample 60529 (NASA). **c** Large angular boulders on the surface of asteroid 101955 Bennu (OSIRIS-REx/NASA/Arizona State University). **d** Dust-mantled rounded particles on the surface of Mars taken by MAHLI on Mars Science Laboratory (MSL) sol 531 (NASA/JPL-Caltech/MSSS)

The size and morphology of regolith particles (Fig. 3) are closely tied to the evolutionary processes that shape a planetary surface, such as impacts (e.g., Hörz 1977; Housen et al. 1979; Ballouz et al. 2020), thermal processing (e.g., Dombard et al. 2010; Delbo et al. 2014; Attree et al. 2018; Lucchetti et al. 2024), weathering, and erosion (e.g., Ehlmann et al. 2008). The particle size frequency distribution (SFD) has been studied for many planetary surfaces, in particular the slope of the SFD has revealed the extent of processing such as impacting, breaking, size sorting, and transporting that the surface materials have experienced (e.g., Carrier et al. 1991; Golombek et al. 2021; Li et al. 2017; Thomas et al. 2001; Michikami et al. 2008; Jiang et al. 2015; Michikami et al. 2016; Burke et al. 2021; Tancredi et al. 2015; Pajola et al. 2024). These evolutionary processes also leave distinct morphological features on boulders across various scales (e.g., Viles 2001), offering valuable insights into the geological history of the planetary body (Yingst et al. 2007; Michikami et al. 2016; Robin et al. 2024). Surface boulder morphology has been studied on various planetary bodies, including Mars (Yingst et al. 2007), comet 67P/Churyumov-Gerasimenko (Cambianica et al. 2019), and several near-Earth asteroids (Michikami et al. 2008, 2010, 2019; DellaGiustina et al. 2019; Michikami and Hagermann 2021; Jawin et al. 2023; Robin et al. 2024).

Previous studies of Phobos using visible images taken during flybys have measured surface boulders in the range of 2 - 85 m (Thomas et al. 2000), but the camera was not able to resolve smaller particles. Therefore, the estimated size of particles at the surface of Phobos, based mostly on the (low) thermal inertia (Lunine et al. 1982; Kührt et al. 1992), varies from tens of microns (Lunine et al. 1982) to several centimetres (Gundlach and Blum 2013). From these observations, the range of particle sizes on Phobos is assumed to be 30 μm–10 cm for the MMX mission (Miyamoto et al. 2021).

The spatial resolution of the TENGOO instrument, onboard the main MMX spacecraft, is 0.3 m at an altitude of 25 km (Kameda et al. 2021). The IDEFIX navigation cameras (NavCams) have a pixel resolution of 0.6 - 0.9 mm at a distance of 1 m (Vernazza et al. 2024). The WheelCams, however, have a pixel resolution of 100 μm at 30 cm distance (Table 2). Therefore, it will be possible to use the WheelCam high-resolution images to measure the particle size distribution down to 200 μm, and to determine the morphology of the surface grains (Hryciw et al. 2016).

Combining the WheelCam-derived particle size frequency distributions with local high-resolution Digital Terrain Models (DTMs) from the NavCams, as well as images and global DTMs from the main MMX spacecraft (Kuramoto et al. 2022) will allow the particle size distribution of Phobos’ regolith to be determined over a far broader range than previous studies (Thomas et al. 2000). It should be noted, however, that given the safety concerns with respect to landing and driving in blocky terrain, the landing site of the IDEFIX rover is likely to be biased towards smoother, finer-grained materials.

#### Cohesive and adhesive properties of regolith particles

Regolith on airless bodies across the Solar System shows evidence of a wide range of cohesive strengths, ranging from quite weak (e.g., near zero on the asteroid 101955 Bennu; Walsh et al. 2022) to quite strong (e.g., more than 1 kPa on the Moon; Heiken et al. 1991). The variation in the cohesive properties of the regolith on Phobos will influence the interaction of the IDEFIX rover with the surface (e.g., the depth of wheel tracks; Sect. 2.2). Additionally, the strength of the cohesion will influence the evolution of the remotely-observed surface. Phobos’ surface exhibits linear geological structures–grooves—that have been linked to the tidal forces coming from Mars (Hurford et al. 2016). Prior modelling work by Cheng et al. (2022) has shown that the structure of the grooves on Phobos’ surface is influenced by the cohesive strength of a subsurface layer. Additionally, Ballouz et al. (2019) considered the gravitationally-induced downslope motion of regolith particles as a possible formation mechanism for the blue units of Phobos. The mechanism described in Ballouz et al. (2019) requires subtle motion of individual particles to reveal surfaces that have not been space weathered. Non-negligible levels of grain-grain cohesion will influence the mobility of single particles, as well as the likelihood that an aggregate (or clump) of particles will move. It is also possible that cohesive forces not only influence the mobility of particles but actually dominate the behaviour of small (< cm-sized) regolith particles on the surface of Phobos (Scheeres et al. 2010).

Regolith clumps have been observed on the Moon by the Chang’E-4 mission’s Yutu-2 rover (Ding et al. 2020, 2021a). Clumps of ice-free regolith particles have also been observed by the COSIMA instrument on the Rosetta spacecraft (Hilchenbach et al. 2017). Both coherent particles and agglomerate clumps in the 10–100 μm size range were observed (Hilchenbach et al. 2017; Langevin et al. 2016). In fact, analysis of COSIMA data showed that only 15% of the dust objects larger than 100 μm were coherent particles, with the majority being clumps (Langevin et al. 2016). Regolith on Mars has also been seen to form clumps, although this may be influenced by the ice and atmosphere on that planet (Lorenz 2022).

**Fig. 4:**
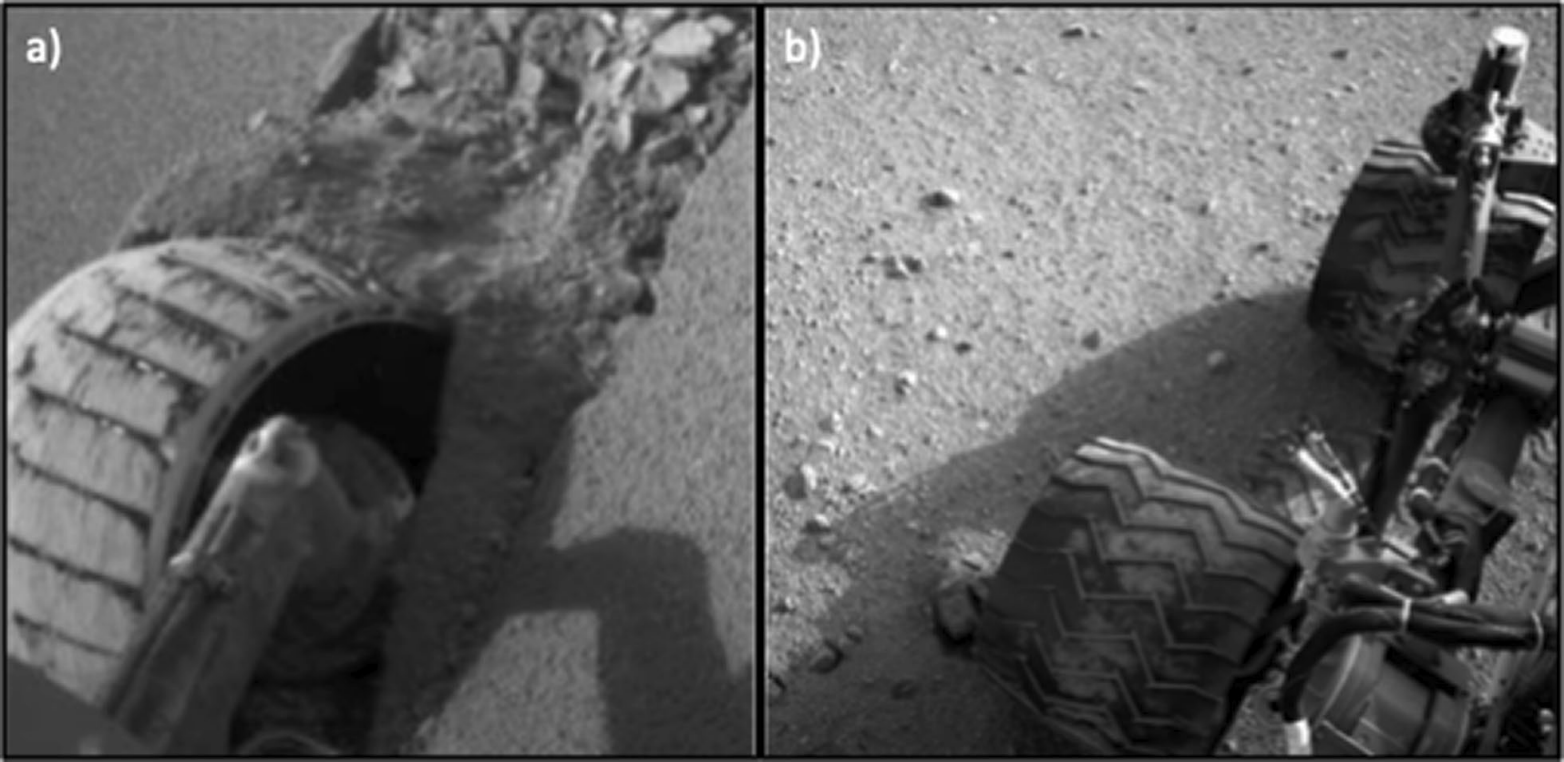
Regolith–wheel interactions. **a** Image taken by Opportunity while attempting to extract itself from Purgatory megaripple. The wheel penetrates deeply into the regolith and fines can be seen adhering to the rover wheel, demonstrating a very cohesive nature of the regolith. Cohesive clumps can also be seen inside the wheel trench (1f170714026esf55pcp1244l0m1.img.jpg; NASA/JPL-Caltech). **b** Cohesive regolith attached to the wheel of the Curiosity rover as observed by the NavCam onboard NASA’s Mars rover Curiosity on Sol 22 (NASA/JPL-Caltech)

The WheelCam images will allow us to detect and characterise the size and shape of regolith clumps in order to obtain cohesive properties of the regolith particles. Images of regolith particles stuck to the WheelCam shutters (after landing and before shutter opening), or to the IDEFIX wheels will also provide constraints on the adhesive properties of the regolith (i.e., the minimum adhesive force required for the grains to remain attached to the surface under Phobos’ gravity). Similar analysis have been performed in the past to quantify the minimum adhesive force between Martian dust particles and the InSight solar panels (Lorenz et al. 2021). Regolith clumps may also be visible on the surface of Phobos, in the tailings behind the wheel and in the rover tracks, as previously observed on Mars by the Opportunity rover (Fig. 4; e.g., Arvidson et al. 2003).

### Bulk mechanical properties of the regolith

The current understanding of the bulk properties of Phobos’ regolith comes from remote sensing and interpretations of surface features (e.g., Kolano et al. 2024). Phobos’ surface is thought to have a low near-surface density and a high porosity based (30–60%) on the very low radar albedo (Busch et al. 2007) and the low thermal inertia (Lunine et al. 1982; Kührt et al. 1992) of the surface material. Interpretations of geomorphological surface features such as the Stickney crater (Bruck Syal et al. 2016) and the grooves (Hurford et al. 2016) also suggest a highly porous, low strength material, while spectral analyses (e.g., Fraeman et al. 2014) reveal similarities between Phobos and carbonaceous chondrites known for high porosity and low density.

The bulk properties of Phobos can give clues as to the formation of the Martian moon. For example, a loose, porous, low-strength material may support the theory of formation through re-accreted debris in orbit around Mars (Craddock 2011), or the captured asteroid scenario given the similarities to carbonaceous asteroids (Rosenblatt 2011).

The IDEFIX rover will make the first in-situ measurements of the mechanical properties of the surface allowing the determination of mechanical properties such as friction, cohesion, density and strength.

#### Mechanical properties from particle morphology

The morphological characteristics of the constituent particles, particularly angularity and roundness, are closely linked to a material’s bulk angle of internal friction (Santamarina and Cho 2004; Cho et al. 2006; Yang and Wei 2012; Yang and Luo 2015; Suh et al. 2017; Kim et al. 2019). For instance, an empirical relationship established through laboratory experiments (Suh et al. 2017) connects the bulk internal friction angle with the average roundness of the particles. Robin et al. (2024) applied this relationship to determine the angle of internal friction for boulders on the surface of Dimorphos near the DART impact site (Daly et al. 2023), and conducted a comparative analysis with boulders on other rubble-pile asteroids. Their findings revealed similar friction angle values across these asteroids (average value of 33.5 ± 6.1$^\circ$), consistent with numerical simulations (Zhang et al. 2022; Raducan et al. 2024), surface slope studies (Lauretta et al. 2022; Barnouin et al. 2019, 2024), and observations of boulder on boulders (Barnouin et al. 2024).

#### Regolith bearing capacity from rover penetration depth

**Fig. 5:**
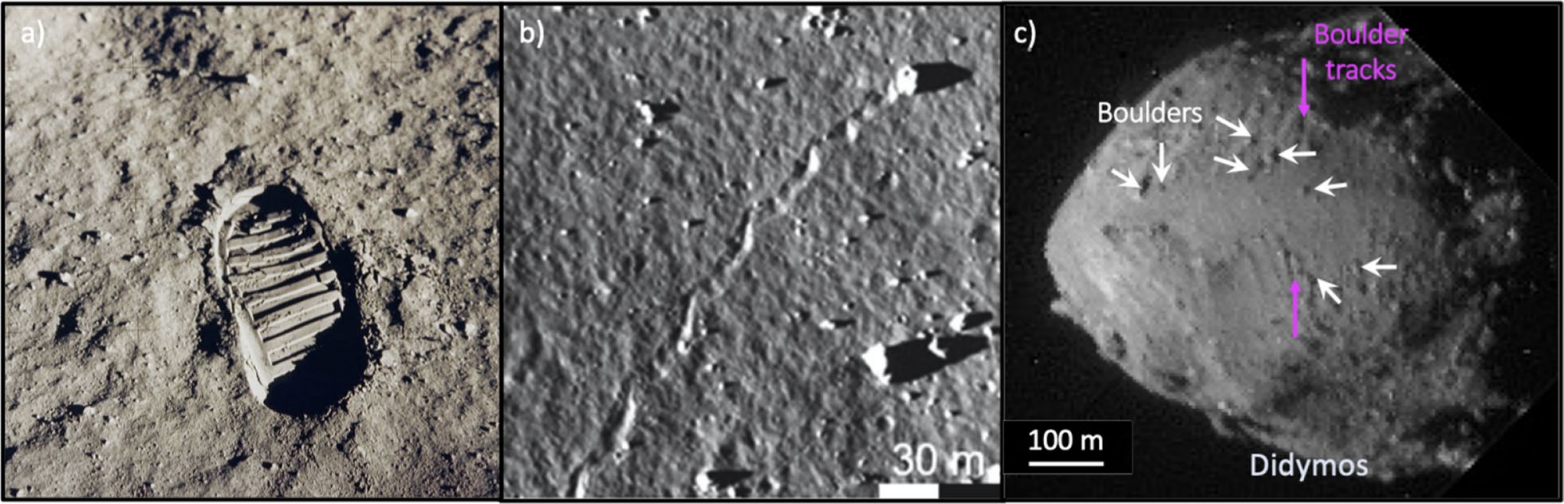
Methods for estimating the bearing capacity of planetary surfaces. **a** Buzz Aldrin’s bootprint from the Apollo 11 mission in 1969 (NASA). **b** Boulder tracks on a pyroclastic deposit on the lunar surface. Image from Bickel et al. (2019). **c** Boulder tracks on asteroid Didymos as observed by DRACO on DART (NASA/APL/ISAE-SUPAERO; Bigot et al. 2024)

**Fig. 6:**
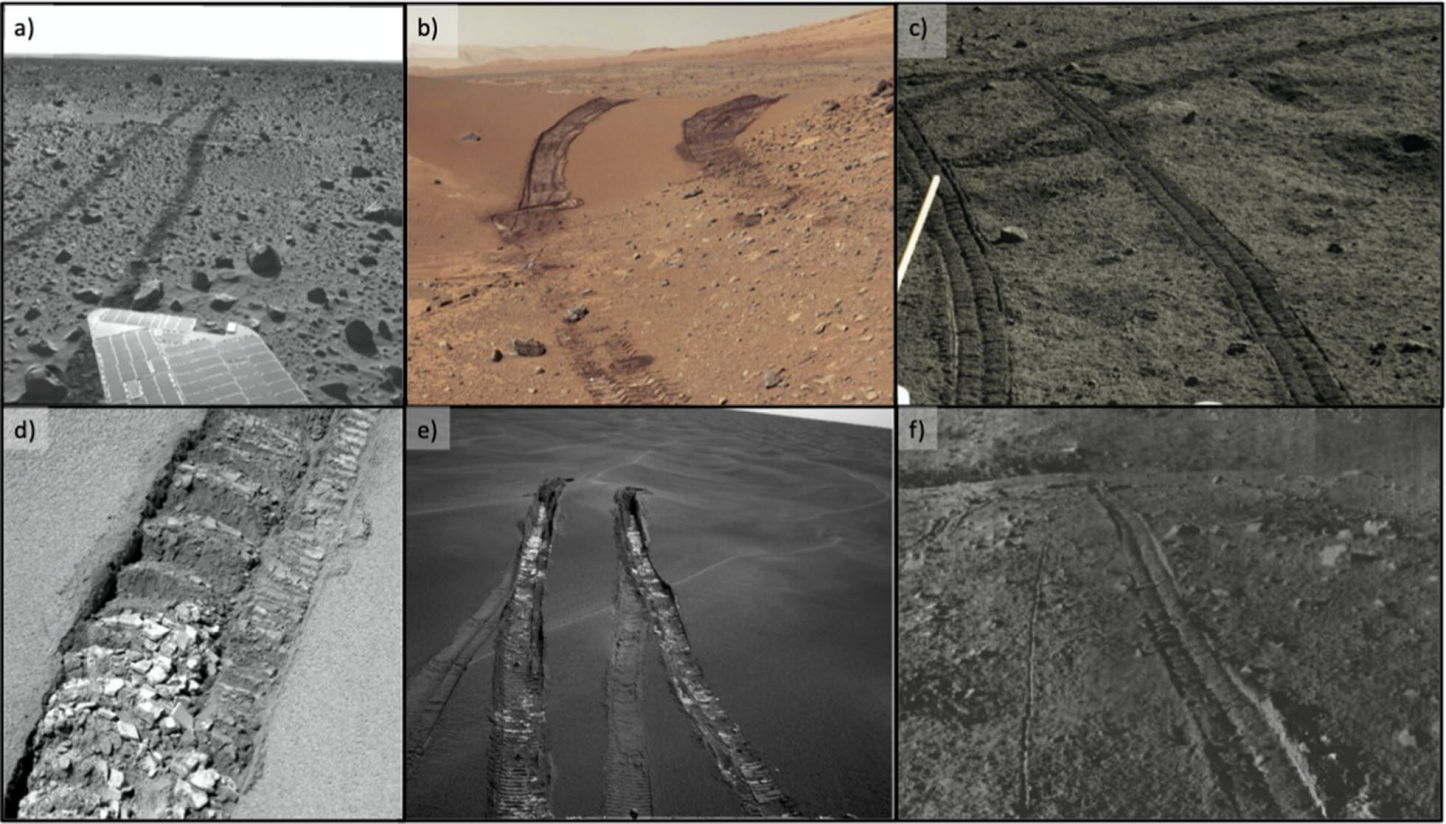
Rover tracks on planetary surfaces. **a** Spirit Rover Navcam image showing shallow rover tracks on Mars. The tracks appear dark as the bright dust cover has been disturbed by the rover (sol 127, image 2N137650599MRL4800P1846R0M1; NASA/JPL-Caltech). **b** Curiosity Color Mastcam mosaic showing tracks on Mars. Tracks on the bedform expose a dark subsurface material, whereas tracks on the rocky surface expose much less dark material (image PIA17944; NASA/JPL-Caltech-MSSS).**c** Yutu-1 rover tracks on the Moon (Chinese national space agency, CNSA; Chinese Academy of Sciences, CAS). **d** Opportunity rover PanCam image showing deep tracks on Mars. The brighter materials are more cohesive, leading to a better perseverance of the wheel treads and the formation of cohesive clumps (sol 447, image F4$\_$1P167868006EFF55DIP2408L4M1; NASA/JPL-Caltech/Cornell). **e** Opportunity NavCam image showing the bright material subsurface material inside the tracks. The second set of tracks that are visible are more dust-mantled and were formed during the approach to the area 6 weeks prior to this image being taken (sol 491, image PIA07999; NASA/JPL-Caltech/Cornell). **f** Lunokhod-1 rover image showing the rovers’ tracks on the Moon. The thin line (between the two tracks) is the 9th wheel, used by the rover to measure the distance travelled (image L1$\_$D03$\_$S01$\_$P02m, Soviet)

The ultimate bearing capacity, or load-bearing strength, represents the maximum pressure that a surface can endure before undergoing shear failure (Meyerhof 1951). This capacity is crucial for assessing whether the surface of a planetary body can support the weight of a lander, rover, instrument, or astronaut. Additionally, it serves as an indicator of the surface material’s trafficability, meaning its ability to provide sufficient traction and propulsion (Bekker 1956; Eggleston et al. 1968; Moore 1970). The bearing capacity of specific regions of the lunar surface has been estimated during the Apollo missions (Carrier et al. 1991). For example, the image of Buzz Aldrin’s bootprint from the Apollo 11 mission in 1969 was used (Fig. 5), along with the weight of the astronaut, to calculate the bearing capacity of the lunar surface (see Bickel et al. 2019, and references therein). The bearing capacity of the lunar regolith was studied from the images of the wheel tracks as part of the Lunokhod-1 and Lunokhod-2 missions (Fig. 6). From these analyses was the bearing capacity of the lunar regolith was found to range from 10 to 100 kN/m$^2$ (Johnson and Carrier III 1971; Slyuta 2014; Basilevsky et al. 2021). The same approach has also been used to analyse the Yutu and Yutu-2 rover tracks (Fig. 6) leading to an estimated bearing capacity of the lunar regolith of 10–20 kN/m$^2$ (Basilevsky et al. 2021). Boulder tracks have also been used to provide bearing capacity estimates, first on the surface of the Moon (Fig. 5; Eggleston et al. 1968; Moore 1970; Hovland and Mitchell 1973; Bickel et al. 2019) and recently on the surface of the asteroid Didymos (Fig. 5; Bigot et al. 2024). These first estimates of the bearing capacity of the surface of an asteroid found a value 1000 times less that that of the surface of the Moon.

The sinkage of the IDEFIX rover wheel, and the depth of the wheel tracks are two independent measurements of the penetration depth of the rover into the surface of Phobos. These rover wheel penetration depth measurements will be combined with geotechnical equations (such as the Terzaghi equation;Terzaghi 1943), in order to determine the bearing capacity of the surface of Phobos.

#### Regolith properties from the rover tracks

**Fig. 7:**
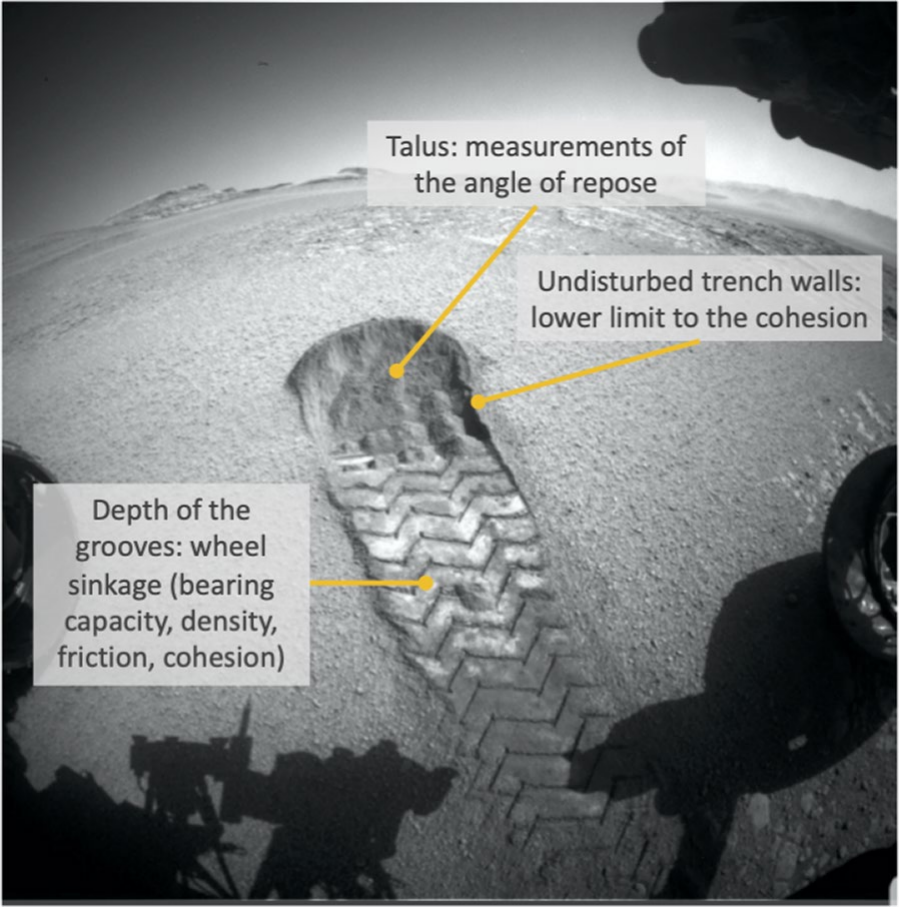
Extracting information about the regolith properties from rover tracks. The example track show here was made by the Curiosity rover (NASA)

Observations of the rover tracks (Fig. 6), talus and tailings behind the wheels can provide additional constraints on the physical properties of the regolith (Fig. 7). The depth of the tracks provides an additional measurement of the rover sinkage that can be used to constrain the bearing capacity (see above) and the density, friction and cohesion of the regolith material (e.g., Carrier et al. 1991; Mitchell et al. 1972). The slope of the talus (i.e., the build-up of loose debris in the track) provides the angle of repose. In the case where the cohesive bonds were broken during talus formation, the angle of repose may also be taken to represent the angle of friction. The undisturbed trench walls provide a lower limit to the regolith cohesion (Sullivan et al. 2011). The volume of the talus is also linked to the regolith cohesive properties with more talus indicating less cohesion. In the case that the IDEFIX rover can reverse, or turn around, the WheelCams and the NavCams (Vernazza et al. 2024) will work in a complementary fashion to study the rover tracks.

#### Regolith strength and stiffness from wheel-regolith interactions

**Fig. 8:**
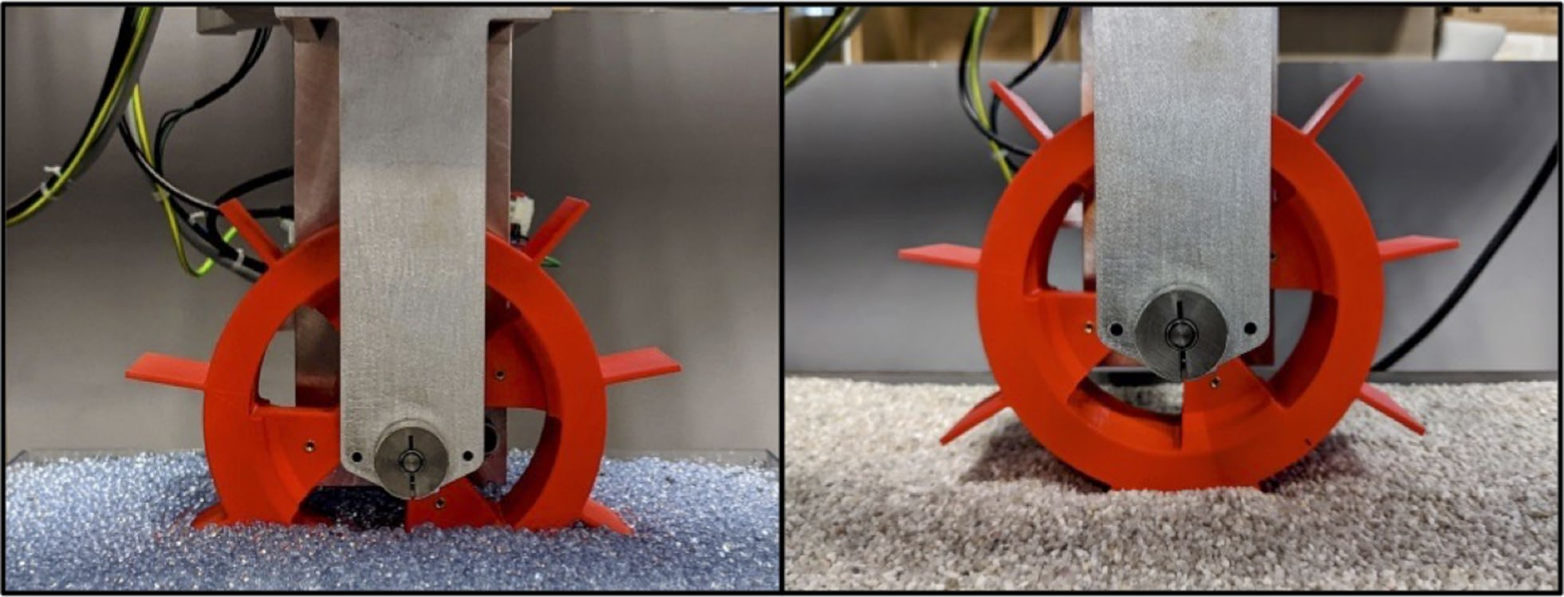
Examples of wheel sinkage into different types of surface materials: (left) glass beads and (right) quartz sand. In both examples the load applied to the wheel is the same but the sinkage is different

The term wheel sinkage refers to the depth to which a wheel penetrates the surface. Sinkage can result from the material beneath the wheel compacting under the vehicle’s load (static sinkage) and from the surface shearing as the wheel rotates (dynamic sinkage). The extent of the sinkage is directly linked to the surface shear strength, which is determined by the bulk density, friction, and cohesion of the surface material (see Fig. 8 and also Sullivan et al. 2011). The wheel sinkage is linked to mobility, as greater sinkage increases the wheel/regolith contact surface area and the resistance to movement.

Rover mobility performance is often evaluated using the slip ratio, which measures the relative motion between the wheel and surface. A slip ratio of 0 indicates perfect rolling, while a slip ratio of 1 signifies the wheel spinning in place without forward movement; a slip ratio of -1 represents skidding, where the wheel moves translationally without rotation. The MER vehicles Spirit and Opportunity, for example, experienced significant slippage, that was highly correlated to the slope direction and magnitude and also to the terrain type (Li et al. 2008; Sullivan et al. 2011). Extensive slippage was also experienced by Curiosity (Rankin et al. 2020).

Previous Mars missions have studied the Martian regolith in detail using the rotation of wheels (Moore et al. 1999; Team 1997; Sullivan et al. 2011). However, many of the techniques applied require measurements of the motor wheel motor currents that are proportional to the torque, or measurement of the electromechanical work (derived from themotor voltages, currents and duration; Sullivan et al. 2011). In the case of the IDEFIX rover, such telemetry will not be available.

Therefore, the rover wheels’ sinkage and slip will be determined using the IDEFIX WheelCam images. These observations will be used to assess the performance of the rover mobility, and also to determine the strength of the regolith (e.g. Sullivan et al. 2011). One method possible is to use the Bekker terramechanics model (Bekker 1956), a semi-empirical approach that assumes that the soil is significantly softer than the wheels of the vehicle. Alternatively, updated versions of the classic Bekker model can use be used e.g., the Reece-Wong model, which includes dimensionless soil parameters and accounts for the dynamic slip-sinkage behaviour of the wheel (Reece 1965), or a model specific to small rovers (Meirion-Griffith and Spenko 2011), or a revised model accounting for wheels with large grousers (Irani et al. 2011). Such approaches have been used to measure the shear strength of lunar regolith from the sinkage of the Yutu-1 rover during the Chang’E-3 mission (Meirion-Griffith and Spenko 2011) and to characterize the regolith at the landing site of the Zhurong rover on Mars (Ding et al. 2021b). Using these approaches, combined with sinkage and slip measurements from the WheelCam images (see Sect. 5.3), the regolith shear strength or stiffness can be estimated. However, the terms in these semi-empirical models depend on gravity. In addition, the low gravity will influence not only the normal force applied on the ground by the rover wheels, but also the behaviour of the regolith itself (Murdoch et al. 2017, 2021), as such the low-gravity environment on Phobos is expected to lead to significantly higher slippage (Kobayashi et al. 2010). Therefore, special care must be taken when applying these models to Phobos and interpreting the IDEFIX WheelCam data (see Section 5.1 and Sunday 2022; Sunday et al. 2022).

### Dynamical behaviour of the regolith

The WheelCam observations will improve our understanding of regolith behaviour on Phobos and granular flow in reduced-gravity environments in general. The WheelCam images can be used to determine the direction and radial extent of the particle motion around the wheel (Skonieczny et al. 2014), which in turn will inform us about the width of the lateral shear interface, the failure characteristics of the surface material, and the thrust of the rover. Measurements of traction and slippage associated with observations of any regolith dilatation around the wheel will also provide information about the shearing characteristics of the regolith. The design of the IDEFIX wheels means that regolith material may avalanche inside the wheel, similar to a rotating drum experiment (see Sect. 5.2 for example images), thus providing direct observations of avalanching regolith on the surface of Phobos. We can also identify clumps of regolith that detach from the surface during rover motion to gain additional information regarding the cohesive properties of the regolith (Sect. 2.1.2).

### Layering in the shallow sub-surface

Small bodies have been known to exhibit sub-surface layering. For example, the artificial crater produced by the Hayabusa2 impact exposed a sub-surface layer of finer particles on asteroid (162173) Ryugu (Ogawa et al. 2022). These results suggest that Ryugu has a layered structure with a different size distribution of the surface layer and the sub-surface layer. Observations from the OSIRIS-REx mission have shown asteroid (101955) Bennu to have a global near-surface layer (1–4 m thick) consisting of finer-grained particles (Bierhaus et al. 2023). The authors predict that such a layer of finer-grained particles should exist on other asteroids depending on the balance between the generation and retention of finer-grained particles. In the same mission a low-albedo dust layer was found to cover the surface of Bennu (DellaGiustina et al. 2019).

In the context of the MMX mission, the proposed reference model for the vertical regolith structure consists of at least three layers: (1) a thin, extremely under-dense uppermost layer (<3 cm thick) of micron-scale accumulated dust; (2) a 10 cm to 3 m thick regolith layer with particles accumulated at relatively high porosity, and (3) a >10 m thick regolith layer with lower porosity (for details see Miyamoto et al. 2021). If layering is indeed present in the very shallow sub-surface of Phobos, the WheelCam images of the IDEFIX rover tracks will allow this to be observed.

### Mineralogy and space weathering of the surface of Phobos

In addition to WheelCam imaging with the white LEDs, images of the surface of Phobos illuminated with three different coloured LEDs (at 590, 720 and 880 nm; see Sect. 3.3) will allow us to perform relative reflectance and albedo analyses that can be used to provide indications of the surface mineralogy. These LED-WheelCam images can be used to reveal the presence of minerals with key absorption bands in the 550–900 nm range [e.g., Clark et al. (1990)], in particular silicates such as olivine and pyroxene that can be detected in the 0.8-1 micron region (e.g., Clark et al. 2020). Such minerals have not yet been identified in the Phobos spectra (Rivkin et al. 2002; Fraeman et al. 2014), but the high spatial resolution of the WheelCams with respect to previous observations may change this.

The blue and red units on Phobos have been hypothesized to be due to different lithologies, or a single lithology affected by different degrees of space weathering (Murchie and Erard 1996) or by different excavation rates (Ballouz et al. 2019). This can be tested by analysing the colour variations in the rover tracks. Depending on the type of planetary surface and the specific terrain, rover tracks can either appear dark due to removal of the lighter dust cover (e.g., Lichtenberg et al. 2007), or light if bright subsurface materials are present (e.g., Zimbelman and Foroutan 2020). Some examples can be seen in Fig. 6. Comparing WheelCam images inside and outside the IDEFIX wheel trenches will allow the Phobos space weathering effects to be assessed. The relative reflectance of the material inside and outside the rover tracks can be compared at the LED wavelengths allowing, for example, for the detection of possible spectral slope changes (e.g., Pieters and Noble 2016). The combined LED-WheelCam measurements will be complementary to the spectral maps in the 450–700 nm range produced by the NavCams (Vernazza et al. 2024).

### Regolith geological classes

Combining all of the above analyses of the regolith particle size and shape, bulk regolith properties, sub-surface layering, mineralogical information and the rover driving behaviour (sinkage and slippage) will allow us to map the regolith along the IDEFIX rovers’ traverse into different geological classes (e.g., drift/loose soil, crusty/cloddy, blocky). This will be similar to mapping that has previously been performed of Martian terrain e.g., by Perserverance (Vaughan et al. 2023).

Scientifically, the geological classes can give indications about understanding surface processes on Phobos. For example, identification of areas with fine-grained particle deposits would imply that there is a regolith transport mechanism on the surface of Phobos, cloddy terrain would indicate the greater importance of cohesive forces in certain regions, and blocky terrain may be an indication of impact generated material. In addition to being of value to the scientific interpretations of the properties and evolution of the surface of Phobos, the resulting classification can be used for informing the rover operations. For example, if a larger sinkage and/or slippage occurred in a particular class of terrain this information can be used to avoid the hazardous terrains in future drives, to adjust the driving speed appropriately, or to increase the performance of the autonomous navigation taking into account the larger slippage.

### Absolute local gravitational acceleration

Knowledge about the gravitational acceleration of Phobos provides insights into its internal structure and its formation and evolution. Phobos has been the subject of extensive investigations to determine its gravitational characteristics and thus its bulk density. For example, telescopic observations and close approaches of Mars Global Surveyor and Mars Express have been used to provide *GM* values in the range of 0.68 to 0.71 $\times 10^{-3}$km$^3$s$^{-2}$ (Lainey et al. 2007; Rosenblatt et al. 2008; Pätzold et al. 2014). These estimates lead to a bulk density estimate of 1850–1860 kg/m$^3$, indicating a significant amount of porosity. However, the second-degree coefficients ($C_{20}$ and $C_{22}$) - that are critical for determining the moments of inertia (e.g., Le Maistre et al. 2019; Matsumoto et al. 2021) - are not currently estimated with sufficient accuracy to place strong constraints on the internal structure (Yang et al. 2019). The geodetic measurements of the MMX mission are expected to significantly improve the accuracy of the gravity coefficients and, in turn, improve the estimates of the moments of inertia in order to detect potential heterogeneities of the mass distribution inside Phobos (Matsumoto et al. 2021; Yamamoto et al. 2024).

It is possible that regolith particles are ejected behind the IDEFIX rover wheel while driving, or that there are regolith particles that fall from the wheel to the ground. Successive WheelCam images (the WheelCams can take up to 6 frames/s; see Sect. 3) will make it possible to track the ballistic descent of the particles to the surface. As there is no atmosphere on Phobos and, therefore, no air drag, the ballistic trajectory can provide an in-situ measurement of the local gravitational acceleration. Such an in-situ estimation of the gravity from images would be a valuable additional data point for the gravitational models. While the main MMX spacecraft may be capable of detecting regional density anomalies (Yamamoto et al. 2024), the in-situ WheelCam - derived estimates could also potentially be used to detect local density anomalies along the rover’s path.

Finally, a more precise knowledge of the gravitational acceleration may be useful for the mission analysis of MMX when operating in the proximity of Phobos, in particular during the landing operations.

**Table 1:** The IDEFIX WheelCam science objectives

WheelCam Science Objective	WheelCam Analysis	IDEFIX Science Objective
(i) Physical properties of regolith particles	Size distribution of regolith particles.	1, 6
	Shape distribution of regolith particles.	1, 6
	Detection and characterisation of regolith clumps to give cohesive properties of the regolith particles.	1, 6
	Detection of regolith stuck to the wheel and/or shutter to give adhesive properties of the regolith grains.	6
(ii) Bulk mechanical properties of the regolith	Mechanical properties (friction) from particle angularity.	1, 3, 6
	Bearing capacity from wheel sinkage/trench depth combined with geotechnical analyses.	6
	Mechanical properties (friction, cohesion) from sinkage and slippage while driving.	3, 6
	Mechanical properties (friction, cohesion) from trench morphology (talus, trench walls).	3, 6
(iii) Dynamical behaviour of the regolith	Determination of the wheel slippage while driving.	3, 6
	Radial extent of particle motion around the wheel while driving.	3, 6
	Analysis of avalanching material inside wheel while driving.	3, 6
(iv) Layering in the very shallow sub-surface	Texture differences, relative reflectance and albedo analyses both inside and outside of the wheel trench.	1, 2
(v) Constraints on the mineralogical composition	Relative reflectance and albedo analyses using images of the surface of Phobos illuminated with different coloured LEDs.	2
(vi) Asses space weathering	Relative reflectance and albedo analyses inside and outside the IDEFIX wheel trench.	7
(vii) Regolith geological classes	Classification based on all available information.	1, 3, 6
(viii) Absolute local gravitational acceleration	Tracking of particles ejected behind the IDEFIX wheel while driving.	3

## WheelCam instrument description

The two WheelCams are placed on the underside of the rover, each looking at a different rover wheel (Fig. 2). Each WheelCam instrument consists of the detector, the optics and a set of co-located LEDs. The WheelCams are protected by a transparent shutter that will be opened after the deployment and uprighting sequence is complete. The WheelCam specifications are provided in Table 2. In this section we briefly describe each of the instrument components and the planned operations on the surface of Phobos.

### WheelCam detector

The WheelCam image sensors make use of a microcamera cube CMV4000 developed by the Centre National d’Etudes Spatiales (CNES), also known as the French Space Agency, and 3DPLUS. This is a generic and multi-purpose camera for Space exploration named CASPEX. The image sensors are panchromatic and consist of a 2048 by 2048 array, with each pixel having a 5.5 μm pitch. A pinned photodiode is used to minimize the noise and achieve a high electro-optic performance. For more information, the reader is referred to Virmontois et al. (2025).

### WheelCam optics

**Fig. 9:**
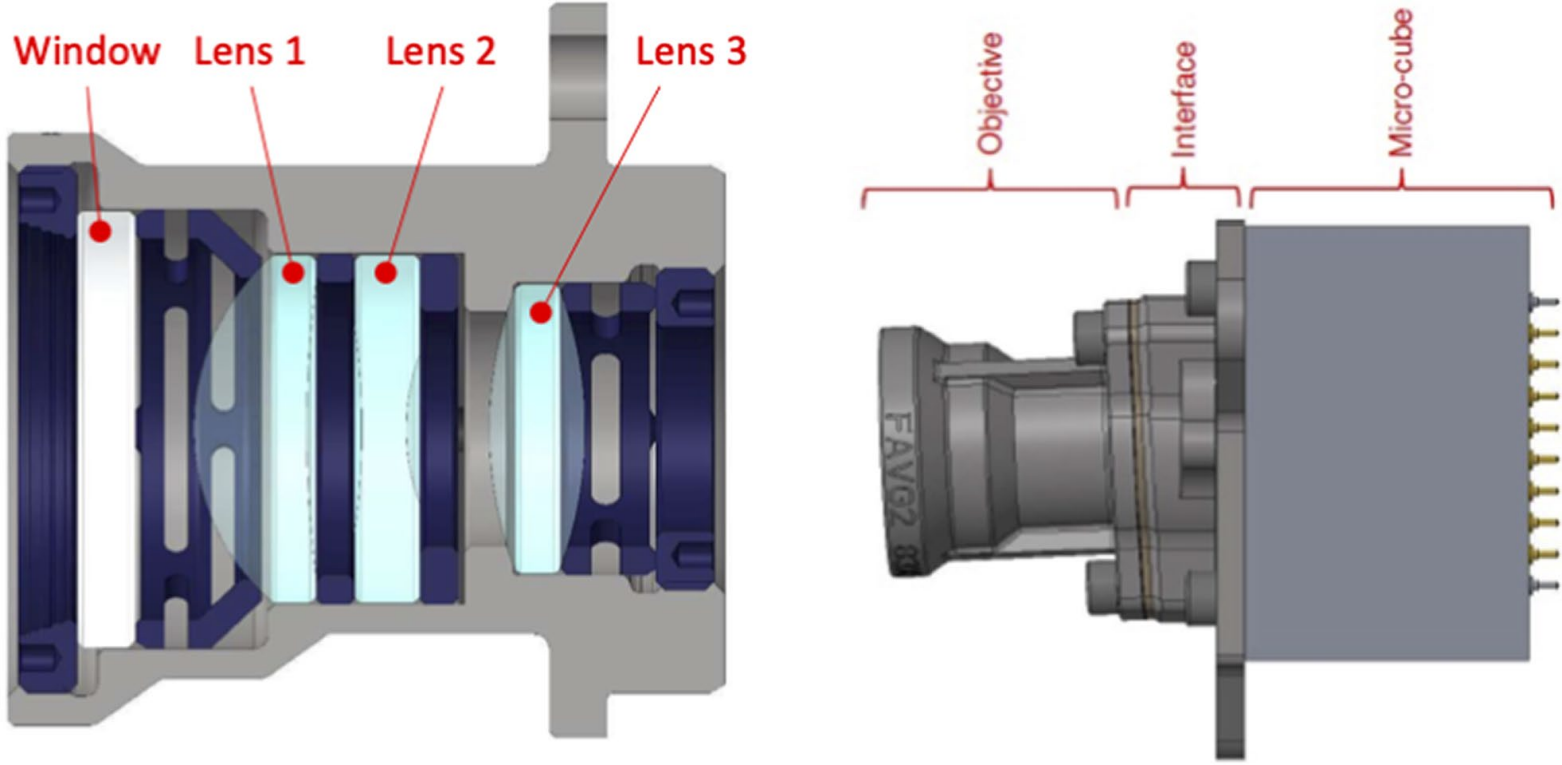
MMX IDEFIX WheelCams. (left) WheelCam optical assembly cross section. (right) WheelCam camera definition. The optics are tilted by approximately 3$^{\circ }$ with respect to the detector

**Fig. 10:**
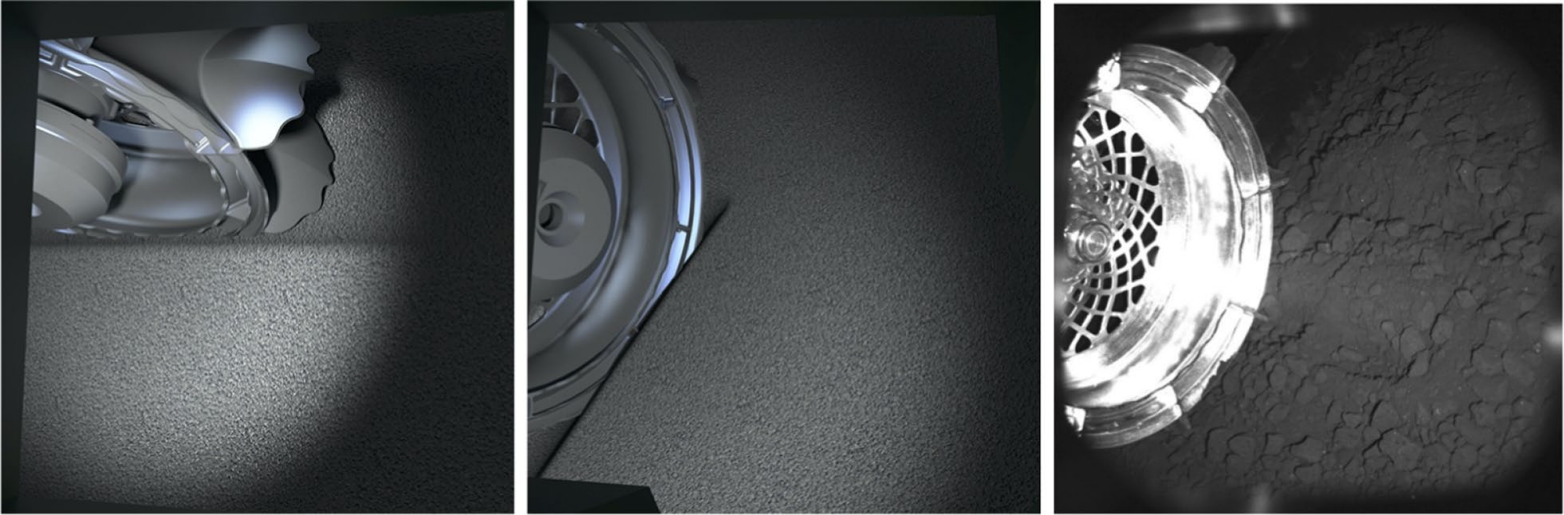
WheelCam field of view. The simulated field of view for the rear (left) and front (middle) WheelCams, and an image (right) from the front WheelCam qualification model taken using the ISAE-SUPAERO WheelCam testbed (see Section 5.2). In the right image the IDEFIX flight wheel is used along with Phobos regolith simulant (Miyamoto et al. 2021)

The optics are provided by OPTSYS (Fig. 9, left). They provide a field of view of 32.5$^\circ$ and a pixel resolution of approximately 100 μm at the center of the image. The WheelCams have an unconventional alignment between the optics and the sensor in order to have the optimal view of the ground (Fig. 9, right). Specifically, to ensure that the entire scene remains in focus, the WheelCam optics are tilted by approximately 3$^{\circ }$ with respect to the detector, with each WheelCam having its own specific tilt angle. As a result, the focus is on a plane that is not perpendicular to the optical axis but positioned near the ground. Although each camera is oriented differently with respect to both the ground and each other, this configuration provides a depth of field of about ±5 cm relative to this plane, ensuring sharp images even when the actual distance to the camera varies between 20 and 50 cm. The simulated field of view of the WheelCams is shown in Fig. 10. In this figure an image taken with the front WheelCam qualification model installed on the ISAE-SUPAERO WheelCam testbed (see Sect. 5.2) is also provided. The dark, shadowed areas around the edge of the images are due to the camera baffles.

### WheelCam LEDs

The field of view of the WheelCams will almost always be in the shadow of the rover’s body and solar panels while on Phobos. Therefore, the WheelCams are also equipped with LEDs to provide illumination. A set of 7 LEDs is integrated next to each camera. These include 4 white LEDs intended for use while driving. Though the white LEDs have enough power to illuminate the scene during short exposure times compatible with driving ($\sim$100 ms), long exposures when the rover is stopped may reveal more details. As the WheelCams are panchromatic sensors, the 3 remaining LEDs have specific bandwidths to allow for multispectral imaging in a focused portion in the center of the field of view. These 3 colour LEDs are the USHIO Epitex L590-09, the USHIO Epitex L720-2AU, and the Epigap EOLD-880-525, with wavelengths of 590 nm, 720 nm, and 880 nm, respectively (Boutillier et al. 2014), which were already space qualified as calibration sources for the EUCLID mission. The white WheelCam LEDs will be used during driving and also to take static images of the ground. The three colour LEDs are intended to be used (alternately) to take WheelCam images while static. An example of the WheelCam LEDs can be seen in the ISAE-SUPAERO WheelCam testbed images below (Sect. 5.2).

**Table 2:** The IDEFIX WheelCam specifications

Field of Vvew (sensor’s edge)	32.5$^{\circ }$
Bandwidth	550–900 nm
Image size	2048 × 2048 pixels
Colour	Panchromatic
Focal length	18 mm
Aperture	F 7.3
Angular resolution	329 μrad
Pixel resolution	100 $\mu$m @ 30 cm
Depth of field	23–38 cm (center of field), tilted
Best focus	30.35 cm
Modulation Transfer Function @ Nyquist	>0.2
Nyquist frequency	91 lp/mm
Pixel pitch	5.5 $\mu$m
Integration time	>193.5 $\mu$s
Read out frequency	up to 6 frames/s (burst), 1.5 frames/s (continuous)
ADC conversion ratio	1 DN = 4.22 e$^-$ (Front), 3.2 e$^-$ (Rear)
Mass	115 g
Power	4 W
Data volume/image	41.9 Mbit (no compression, no binning)

### Planned WheelCam Operations

During the rover Separation, Landing, Uprighting and Deployment (SLUD) phase, the WheelCams are covered by a protective transparent shutter. The WheelCam shutters will only be opened when IDEFIX is in the upright position.

The WheelCams can be operated in both an imaging and a movie mode, the latter being intended to be used during driving. The anticipated IDEFIX rover speed on the surface of Phobos is 0.1-4 mm/s, and typical movie frame rates are expected to be 1 image per mm moved for the front WheelCam and 1 image per cm moved for the rear WheelCam. Each driving sequence is also planned to be combined with at least 8 context images taken by the rover NavCams (Vernazza et al. 2024).

Observations of a full rover wheel rotation will require multiple images. To reduce the data volume transmitted to Earth via the MMX spacecraft, the (2048 × 2048 pixel) WheelCam images can be binned. The binning (2 × 2, generating a 1024 × 1024 pixel image) can be performed directly during the WheelCam acquisition, or post acquisition. In the latter case, it is possible to bin the images multiple times and the original image will remain available onboard at full resolution for some time (depending on how many acquisitions are performed after it was captured). The level of binning requested will depend on the number of images, the available bandwidth, which is mostly constrained by the spacecraft—Earth link, not by the rover—MMX spacecraft link, and the data production of the other instruments of the rover. If image compression is implemented this could be a viable alternative to the binning.

## WheelCam characterisation

**Fig. 11:**
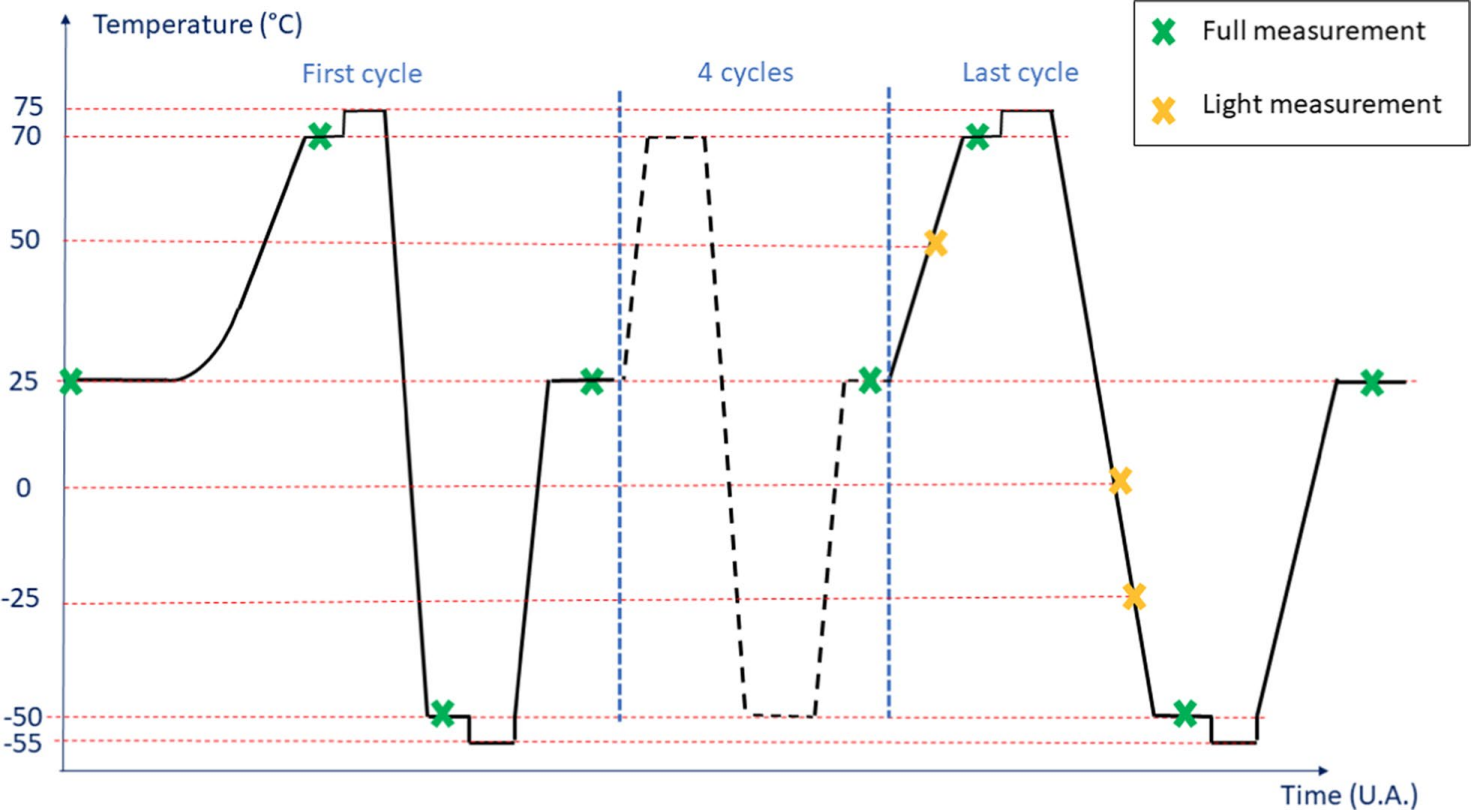
Thermal Vacuum Chamber acceptance test sequence for the WheelCams. During a “Full measurement”: dark, FTEO, resolution, distortion and flat measurements were made. During a “Light measurement”: dark, resolution, distortion and flat measurements were made

The characterisation of the flight model of the microcamera cubes (without the optics) were performed at 3DPLUS. The characterisation of the flight model optical lens assemblies for the front and rear WheelCams were performed at OPTSYS. The integrated WheelCam tests were then performed using the optoelectronic bench located in at CNES inside the ISO7 clean room. The flight models of both WheelCams have been tested in a thermal vacuum chamber with 6 thermal cycles with the electro-optical performances being measured at several temperatures (Fig. 11). The test procedures largely followed those used for other CASPEX cameras at CNES (e.g., Théret et al. 2024; Virmontois et al. 2025).

### Dark measurements

Measurements were performed in darkness for different integration times and at different temperatures (see Fig. 11 for the temperature profiles). From these measurements the dark current, fixed pattern noise (FPN) and readout noise were characterised.

**Fig. 12:**
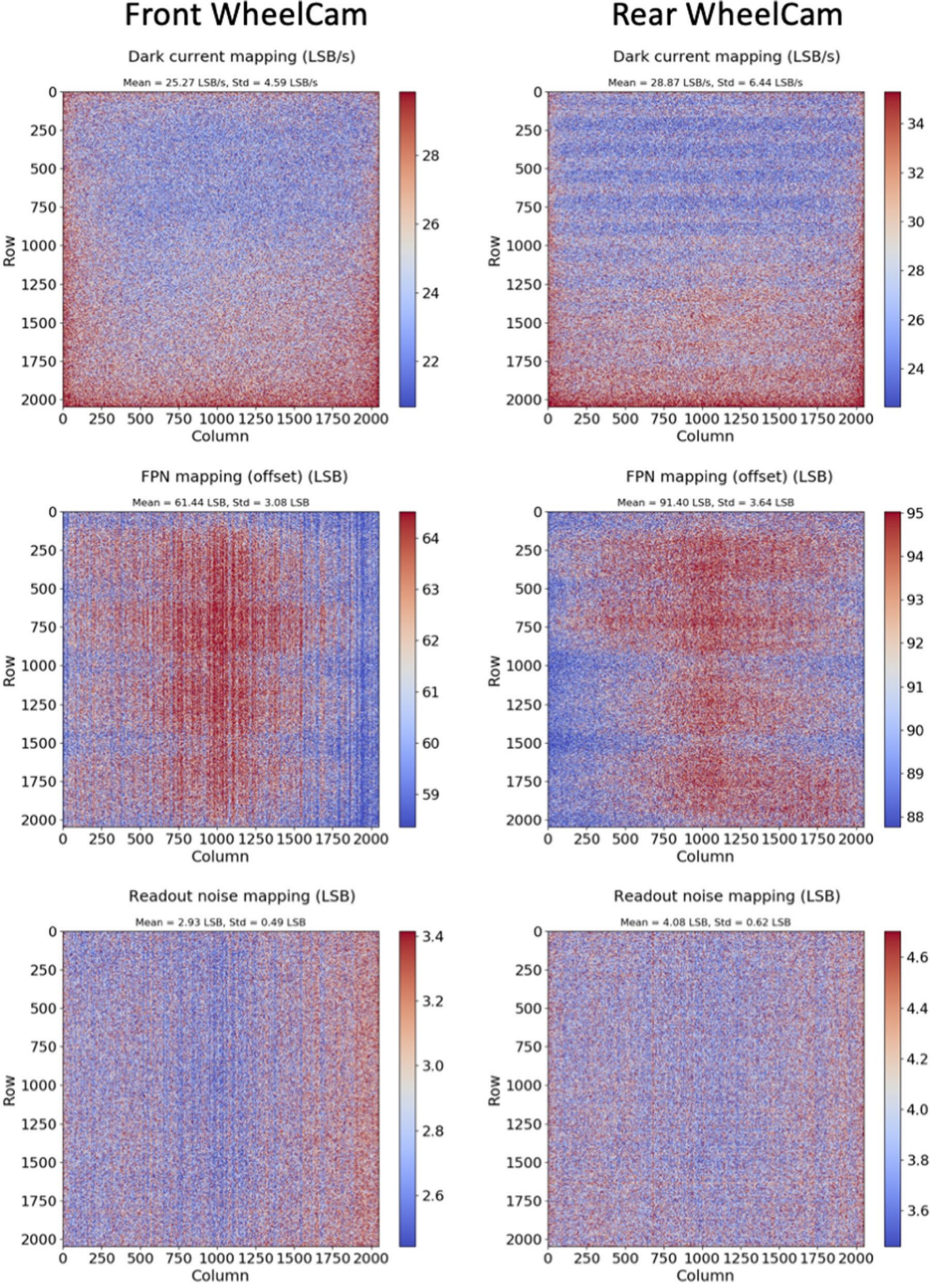
Dark measurements of the WheelCam flight models. Results for the front WheelCam are shown on the left, and results for the rear WheelCam are shown on the right. (Top) Dark current mapping (in LSB/s) at 25$^{\circ }$C. The pixel array is given with a colourbar whose intervals are [$\mu _{darkcurrent} - \sigma _{darkcurrent}$ ; $\mu _{darkcurrent} + \sigma _{darkcurrent}$], where $\mu _{darkcurrent}$ is the average dark current and $\sigma _{darkcurrent}$ is the standard deviation of the dark current. (Middle) The offset or FPN measurement in LSB following the same colourbar rule as the dark current measurement. (Bottom) The readout noise in LSB following the same colourbar rule as the dark current measurement

The dark current is calculated as the slope of the signal curve against integration time (least mean square linear regression) in the dark (no light sources). The offset or FPN measurement represents the average pixel value at the lowest integration time (193.5 μs for the WheelCams) and the readout noise is also determined at the shortest integration time, and represents the temporal standard deviation on a measured pixel value in LSB. Example results of each of these are presented with a mapping of the pixel array in Fig. 12.

From these measurements the dark current temperature dependence was extrapolated over the operating range and was found to be exponentially proportional to the temperature, as expected. The average dark current at 25$^{\circ }$C is 560 e$^-$/s for the front WheelCam, and 203 e$^-$/s for the rear WheelCam and the average readout noise at 25$^{\circ }$C is around 13.8 electrons for the front WheelCam and 13.9 electrons for the rear WheelCam.

### Under-light measurements

The under-light measurements permitted the characterisation of the WheelCams charge to voltage factor (CVF), electro-optic transfer function, resolution and flats.

**Fig. 13:**
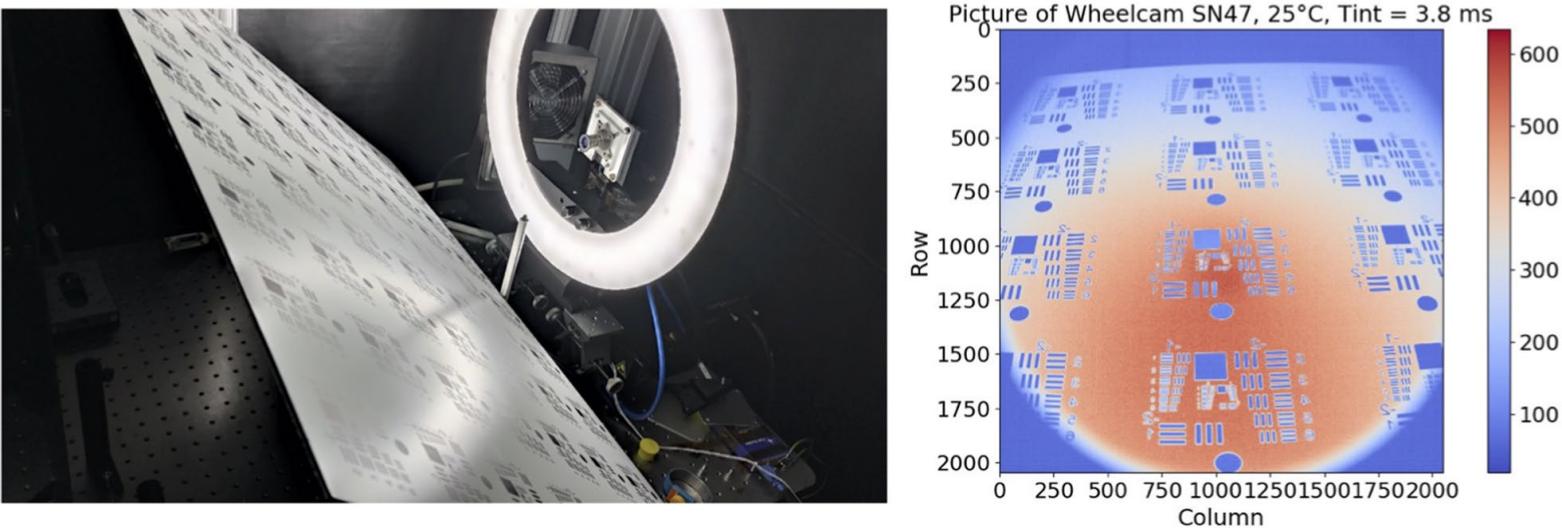
WheelCam resolution measurements. (left) Optical bench for WheelCam resolution measurements. (right) Example for the front WheelCam when the test pattern is at 32 cm. No corrections (radiometric, distorsion) have been applied

For the CVF and the electro-optic transfer function tests the same test bed is used. This test bed makes use of a LED source (SOLIS-3C from Thorlabs) combined with filters to light the detector with monochrome light at 600, 700 and 900 nm. The wavelength of the radiometer is changed at each wavelength to measure the illumination of the source and approximately ten points of integration time have been acquired for all the temperature steps. For the resolution measurement a USAF bar reflective test pattern is placed in front of the sensor at different locations in the field of view (Fig. 13). For each WheelCam, the poster is tilted with different angles at a different distance. The horizontal and vertical bars in the resulting images are used to compute the contrast and the resolution. For the flats, all the surface of the camera’s array is illuminated using a uniform source using an led screen in front of the camera.

The CVF is 0.237 LSB/e$^-$ for the front WheelCam and 0.316 LSB/e$^-$ for the rear WheelCam. The different CVF values lead to different saturation levels for the two instruments (4316 e$^-$ for the front and 3237 e$^-$ for the rear WheelCams). The spatial period (resolution) where the contrast reaches 0.2 is 240 μm for the front WheelCam and 243 μm for the rear WheelCam. There were no defective pixels detected on the front or the rear WheelCam. The thermal cycling did not affect the instrument performance as there was no deviation between the first and the last measurements for the cameras.

### Geometric calibration

**Fig. 14:**
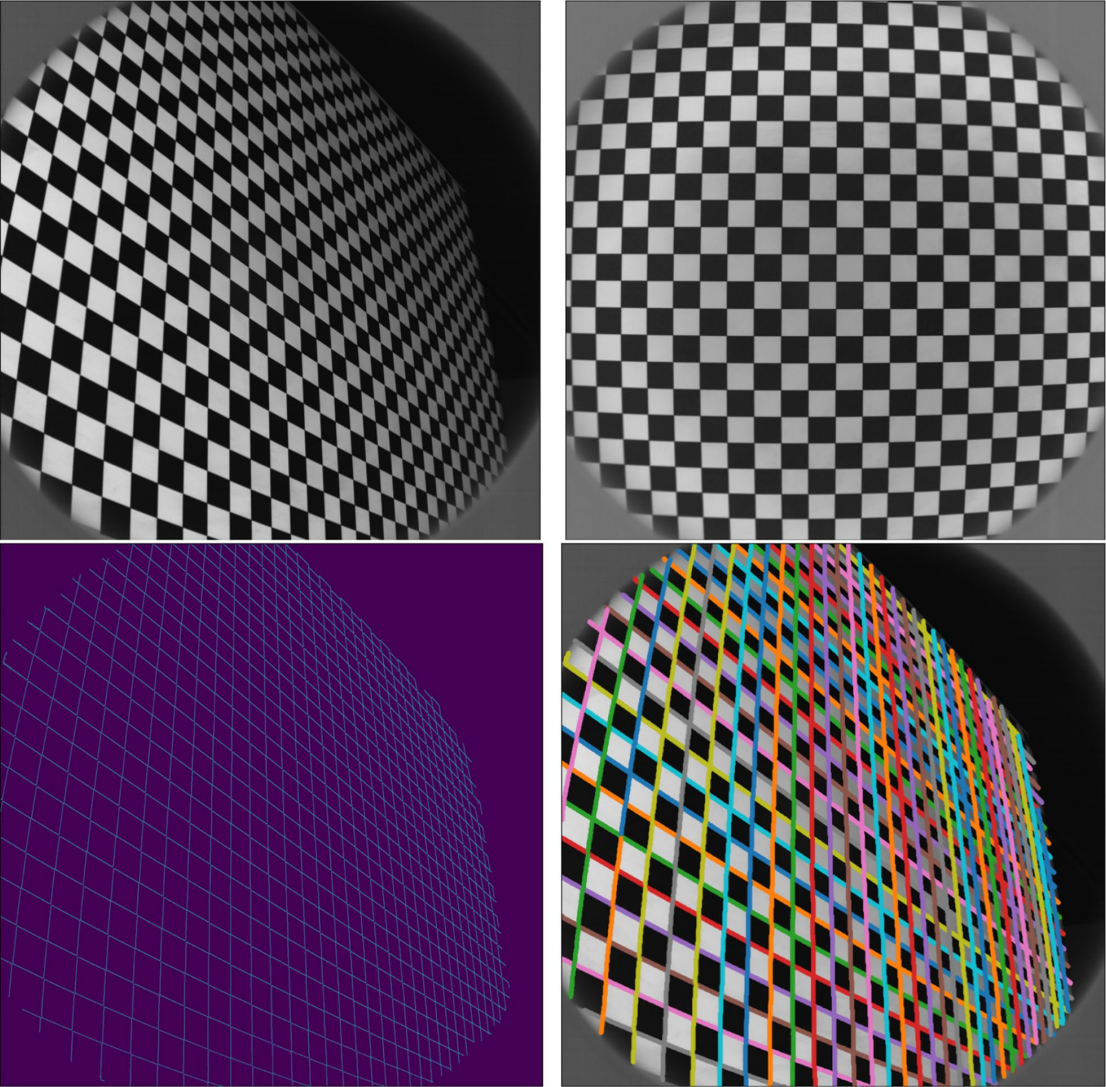
Checkerboard images and gridline detection (Top) Example checkerboard images acquired with a WheelCam. (Bottom) Grid line detection. Left: Canny edge filtering. Right: Line grouping

The distortion is assessed with a checkerboard target, as shown in Fig. 14 (top). Several acquisitions of the checkerboard are done with different orientations, in order to constrain distortion effects and improve the efficiency of the assessment. Checkerboard images have to cover the whole field of view, and several acquisitions are averaged for each position and orientation, so that the Signal to Noise Ratio (SNR) is higher for each resulting image.

For each image, a Canny filter (Canny 1986) is applied first to detect edge coordinates. These points are located along the white/black transitions of the checkerboard. Then the points are grouped into lines using an edge angle parameter and a threshold defined as a maximum Euclidian distance between 2 points (Fig. 14, bottom).

**Fig. 15:**
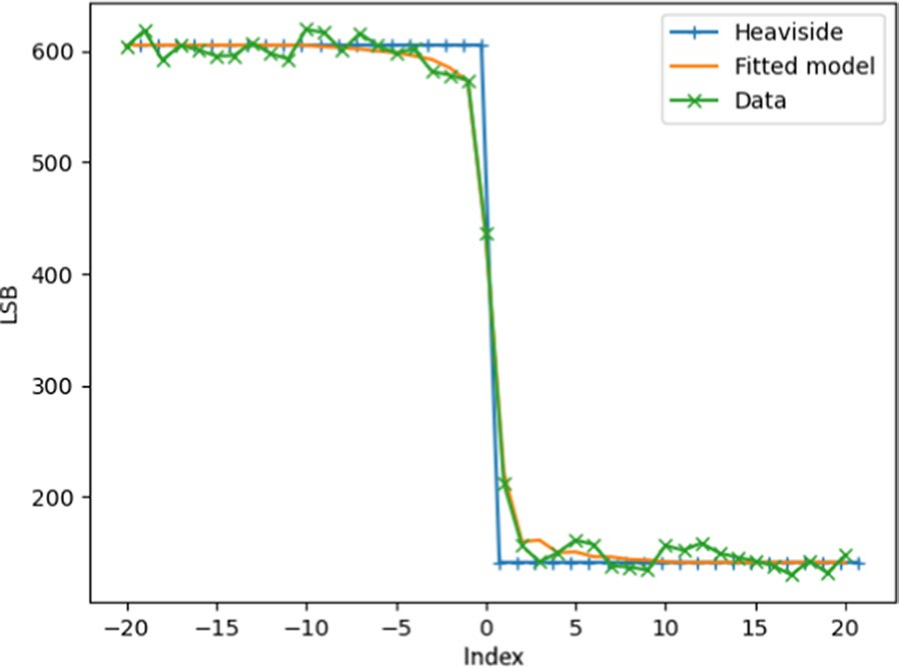
WheelCam radiometric transition model. Green: Single edge transition. Blue: Heaviside function. Orange: Convoluted Heaviside function

To improve the performance, the edge coordinates are refined with an edge radiometric transition model:

- First, the radiometric transition is extracted across the considered edge (green curve in Fig. 15).
- Then this transition is modelled with a Heaviside function convoluted by the camera’s Point Spread Function (PSF) (blue and orange curves in Fig. 15).
- Finally, a least square minimization is performed to find the best edge coordinate (Fig. 15), and deduce the accurate positions of the full lines.

The quality of the checkerboard must be high enough to cope with the camera’s spatial resolution. In the WheelCam case there was a residual default in the checkerboard printing that increases the error in the optimization (white and black transition are not perfectly aligned), so it was necessary to average the errors with a distortion model and estimate the residuals of the model with respect to the acquired data.

The next step is to match the refined points with a grid model in order to extract the distortion model. The grid model, which represents the checkerboard, is defined by its total grid size and by the step between the lines, in meters. Each checkerboard configuration, referring to the acquired calibration images, is defined by its position (3 values in meters) and by its orientation, given by a unitary quaternion. Knowing that, the grid is projected on the camera’s focal plane using the focal length and the pixel size, and the distortion model is applied in the focal plane geometry. Several models exist: the simpler it is, the more robust it will be, but a sophisticated model can also give a more precise measurement and therefore a better distortion correction.

Different configurations from the Brown distortion model (Brown 1996) were considered for the WheelCams. Based on the RMS error between the model and the measurements for different models and different configurations (number of radial coefficients), it was decided to implement the following distortion model with three radial (*r*), two tangential (*t*) and four thin prism (*p*) coefficients:

$$\begin{pmatrix} x'\\ y' \end{pmatrix} = \begin{pmatrix} \begin{array}{l} x + (x-x_c) \cdot (r_1d^2+r_2d^4+r_3d^6) \\ \quad + t_1(d^2+2(x-x_c)^2) + 2t_2(x-x_c)(y-y_c) \\ \quad + p_1d^2 + p_2d^4 \\[1ex] y + (y-y_c) \cdot (r_1d^2+r_2d^4+r_3d^6) \\ \quad + t_1(d^2+2(y-y_c)^2) + 2t_2(x-x_c)(y-y_c) \\ \quad + p_3d^2 + p_4d^4 \end{array} \end{pmatrix}$$

where (*x*, *y*) are the original coordinates on the image, while ($x^\prime$, $y^\prime$) are the corrected ones. $d = \sqrt{(x-x_c)^2 +(y-y_c)^2}$ is the radial distance from the center of the distortion $(x_c, y_c)$, $r_1$, $r_2$, $r_3$, $t_1$, $t_2$, $p_1$, $p_2$, $p_3$ and $p_4$ are fixed parameters corresponding to the barrel distortion.

The lines describing the distorted grid model then have to be linked with the measured lines. To do this, and because the distortion is quite small, the grid model parameters are roughly estimated for each checkerboard configuration. Then, the measured lines are linked with the lines from the model by minimising the square of the distance between them. Once the lines are linked, a least square minimization (LMFIT python library with Levenberg-Marquardt method) is used to match the measurements with the model. The computed parameters are the parameters of the distortion model, as well as the parameters of the grid model.

**Fig. 16:**
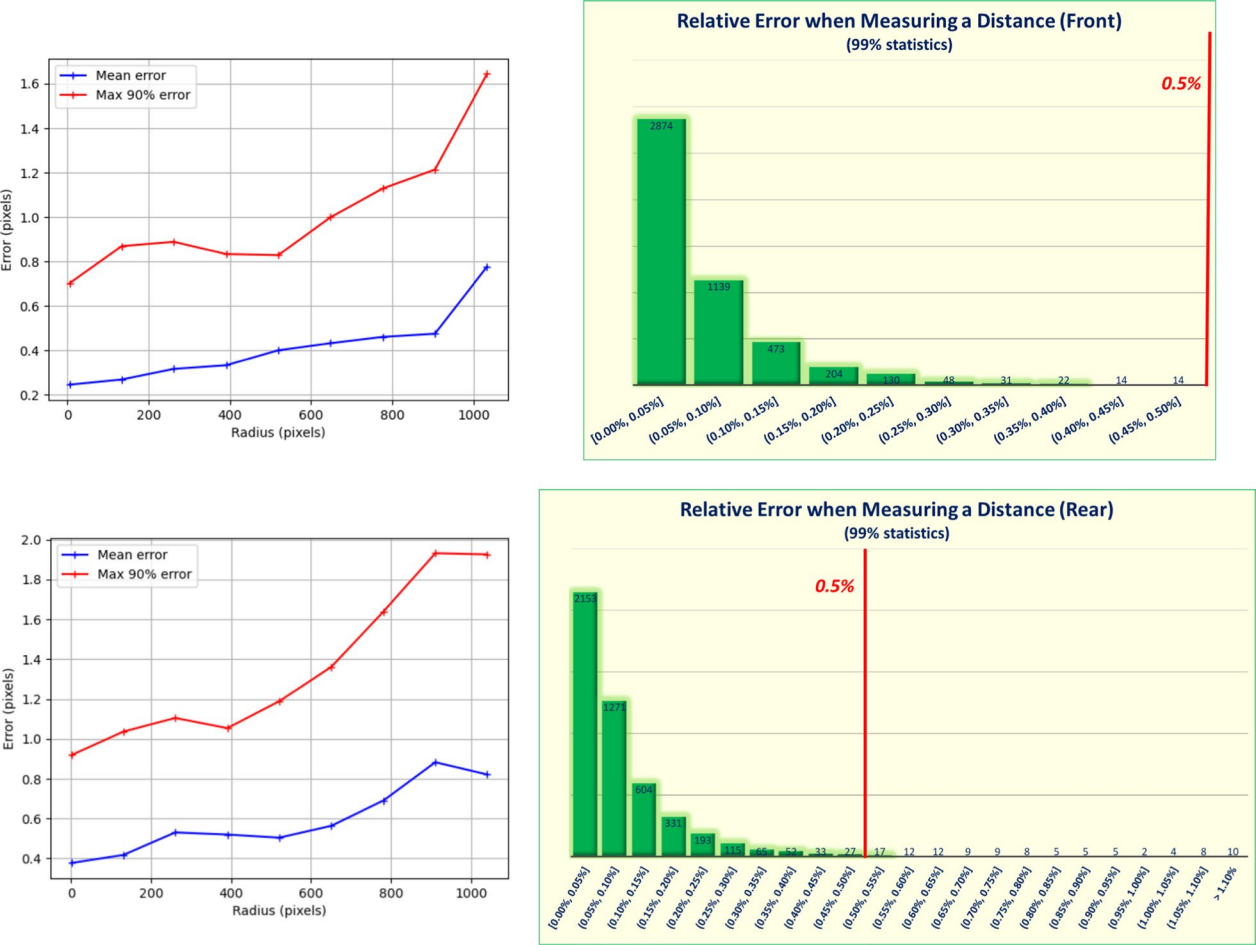
WheelCam distortion errors. Left: Front (above) and Rear (below) WheelCam Geometrical distortion model error. The radius is the distance from the center of the image i.e., ($x_c$, $y_c$). Right: Front (above) and Rear (below) WheelCam relative error when measuring a distance

Finally, the difference between the model and the real images is computed in order to estimate the error of the distortion correction. This error depends mostly on the distance from the center of the image and is illustrated in Fig. 16 (left) for the rear WheelCam. However, the most relevant assessment is to estimate the relative error (in percent) which is made when measuring a distance with a rectified WheelCam image, due to the distortion. This error is given in Fig. 16 (right) for the WheelCams and is less than 0.5% 99% (front) and 97% (rear) of the time, and less than 0.05% 61% (front) and 46% (rear) of the time.

## Preparing for the analyses and interpretation of WheelCam images

In order to prepare for the analysis and interpretation of the IDEFIX WheelCam images, we have developed both numerical simulations and an experimental single wheel testbed. These tools are complementary, increasing our understanding of how the wheel-regolith interactions are likely to change in the Phobos gravity environment, and allowing representative images to be generated in a terrestrial environment, respectively. In the following, we describe the current status of each of these tools and the data processing methodologies have been developped.

### Numerical simulations of sinking and rolling

Simulations are significantly more complex and expensive to run than any analytical models. Nonetheless, they play a crucial role in evaluating rover behaviour within environments that cannot be replicated on Earth. This is also essential to ensure an accurate determination of the regolith properties from the wheel—surface interactions. Numerous rover studies have used numerical techniques such as the finite element method, discrete element method, material point method (Agarwal et al. 2019), the dynamic Bekker method (Smith et al. 2014) and the Soil Contact Model (SCM; Schäfer et al. 2010; Tasora et al. 2019; Krenn and Gibbesch 2011).

**Fig. 17:**
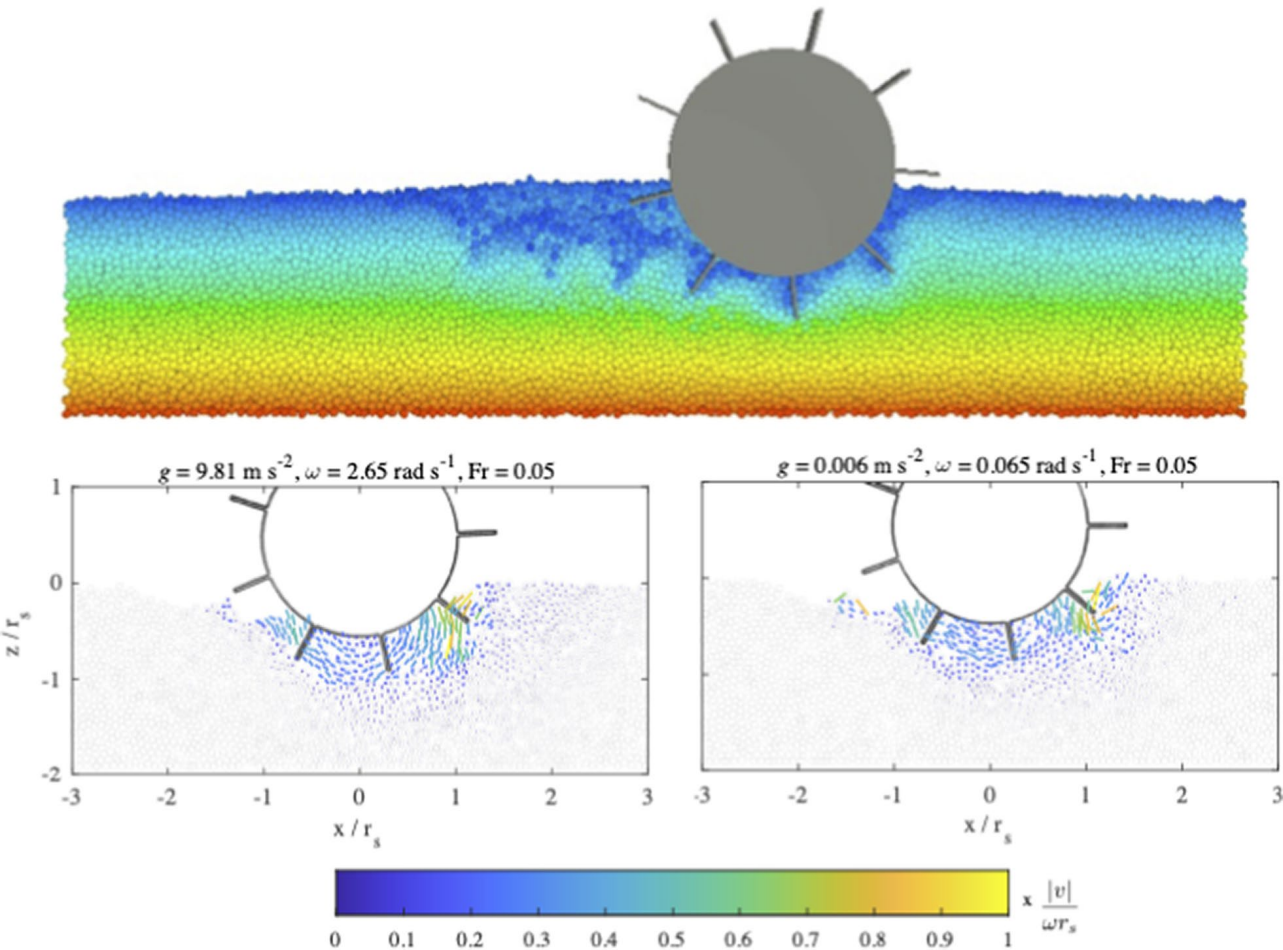
Discrete Element Method simulations of rolling in Earth and Phobos gravity. (Top) Snapshot from a rolling simulation in terrestrial gravity with a wheel rotational velocity of 0.65 rad/s. The container is filled with 6 ± 0.5 mm diameter rough spherical particles. The particles are coloured by their vertical positions at the start of the simulation. (Bottom) Visualisation of the particle flow around the rover wheel after a 90 degree turn for simulations with different two different gravitational accelerations (left—Earth, right—Phobos), but the same rotational Froude number (0.05). The particle velocity vectors are coloured by $v/\omega r_s$, where $r_s$ = 77 mm, *v* is the velocity magnitude of the fastest moving particle, and $\omega$ is the rotational velocity of the wheel. For further details see Sunday (2022)

In order to understand the influence of the low-gravity environment on sinking and driving behaviour we have performed soft-sphere discrete element method simulations (Sunday et al. 2020). We have conducted simulations to analyse the sinkage, the slip, and the driving distance for a simple rover wheel under Earth and Phobos gravity conditions for different simulated terrains and different wheel rotational velocities (see Fig. 17; Sunday et al. 2022; Sunday 2022). These previously reported simulations demonstrated that the performance of the MMX rover will largely depend on the surface characteristics of Phobos and that at higher driving speeds, the material beneath the wheels will fluidize, leading to wheel slippage and loss of traction. The results of Sunday (2022) also demonstrated that the behaviour of the rover’s wheels likely scales with the rotational Froude number, $Fr = \frac{\omega ^2}{gR}$, where $\omega$ is the rotational velocity of the wheel and $R$ is the wheel radius, in agreement also with the work of Slonaker et al. (2017), who employed resistive force theory to propose scaling relationships for locomotion on granular surfaces.

The Froude number serves as a powerful scaling tool, enabling us to predict behaviour on small celestial bodies based on observations on Earth in the case of equivalent materials on both Earth and Phobos. For instance, if the MMX rover travels at a no-slip velocity of 2.5 mm/s on Phobos, similar sinkage and traction could be observed at a speed of 100 mm/s on Earth, assuming similar but non-cohesive materials.

These simulations will be continued in the lead-up to the IDEFIX operations to extend the parameter space (e.g., varying particle size distribution, frictional and cohesive properties), and evaluate the performance of existing terramechanics models that link the driving performance (sinkage and slippage) to the surface properties. The detailed information available from simulations is also important to deepen our understanding of the physical mechanisms that lead to a different behaviour in a low-gravity environment (e.g., the reduced friction forces and increased flowability of regolith material in low gravity). In addition to providing valuable information for the rover operations about the expected driving behaviour, the simulations will also be used to invert the surface properties based on the surface interactions observed by the WheelCams (similar to the approach presented by Ballouz et al. (2021) to infer the properties of asteroid Bennu’s surface from the OSIRIS-REX touch-down event).

### WheelCam testbed

A single-wheel MMX IDEFIX WheelCam testbed that recreates the scene that the WheelCams will observe during the mission has been developped at ISAE-SUPAERO. The main objective of the testbed is to develop the image processing tools for the WheelCams (see below), but the testbed can also be used to compare wheel sinkage and driving performance for different types of surface materials.

**Fig. 18:**
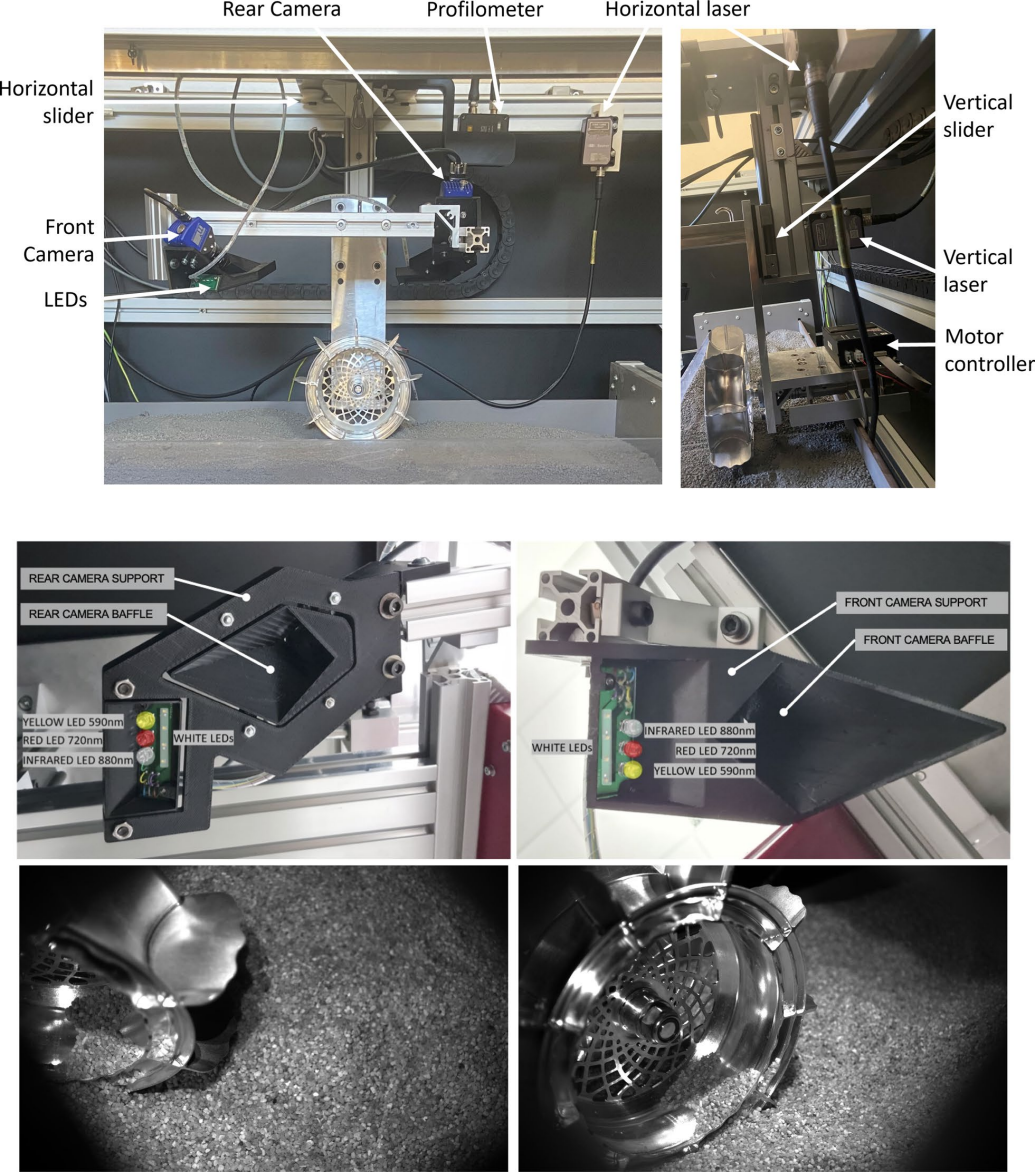
WheelCam testbed at ISAE-SUPAERO. (Top) Detailed view of the wheel assembly on the WheelCam testbed. The Wheel assembly can be lifted and lowered into place using the pulley system shown in the photo. The wheel is driven by a brushed DC motor and can translate freely in the vertical and horizontal directions. (Middle) The WheelCam LEDs as mounted on the ISAE-SUPAERO WheelCam testbed for the rear (left) and front (right) cameras. The camera supports and baffles have been 3D printed for the testbed but their form is the same as on the IDEFIX rover. The LEDs have also been mounted and positioned identically as on the IDEFIX rover. (Lower left) Rear camera perspective. (Lower right) Front camera perspective. These field of views generated by the WheelCam testbed at ISAE-SUPAERO are approximately the same as the IDEFIX WheelCam field of views. The surface material in these images is quartz sand

The testbed (Fig. 18) consists of three main sub-assemblies (the main structure, the material container, and the wheel assembly), multiple sensors (a motor encoder, two displacement sensors, and a laser profilometer), and two cameras. The two cameras are positioned to observe the wheel-regolith interactions from the same perspective as the IDEFIX WheelCams. The actual IDEFIX WheelCams point towards two different wheels, but the testbed cameras point towards a single wheel, reducing the complexity of the setup. The different sensors allow measurements to be made of the sinkage and slippage of the wheel and the trench morphology. The testbed also includes the IDEFIX rover wheel, camera baffles, and the WheelCam LEDs (Fig. 18) in addition to blackout panels to perform trials in representative lighting conditions. Example images from the testbed cameras are shown in Fig. 18. More information can be found in Sunday (2022).

The ISAE-SUPAERO WheelCam testbed has also been used to determine the expected performance of the WheelCams under realistic conditions. Fig. 10 (right) shows an example image taken by the Qualification Model of the front WheelCam installed on the testbed. In this image the exposure time is 200 ms, the regolith is a Phobos simulant (Miyamoto et al. 2021), and the wheel and LEDs are both equivalent to the flight-models. These tests have provided the most representative images of those that can be expected on the surface of Phobos.

### WheelCam image processing

To perform the analyses listed in Table 1 in order to achieve the WheelCam scientific objectives, several image processing approaches will be used. Below we give a non-exhaustive list of some example image processing techniques being developped at ISAE-SUPAERO that will be applied to the IDEFIX WheelCam images. The developped algorithms will be described in detail in future work.

#### Particle size and morphology

**Fig. 19:**
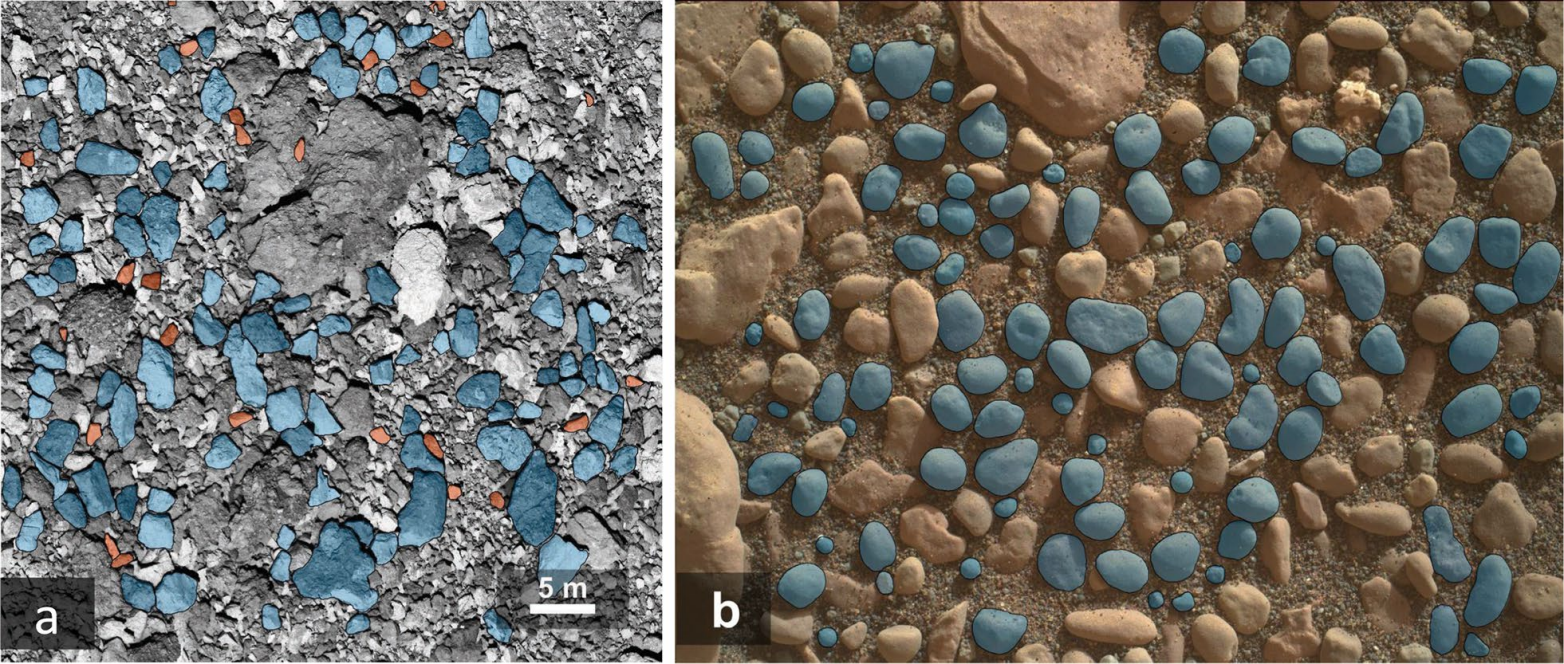
Examples of image segmentation for size and morphological characterisation. Left: Angular particles on the surface of asteroid (101955) Bennu taken by OCAMS on OSIRIS-REx (NASA/University of Arizona). Right: Image of rounded pebbles on the surface of Mars taken by MAHLI on sol 2356 of the Mars Science Laboratory Mission (NASA). The particles detected through the semi-automatic segmentation are used for morphological characterisation are shown in blue. The red particles are less-well resolved (<30 px), and should be not be used for resolution-dependant morphological parameters. For more details see Robin et al. (2024)

A dedicated image-processing pipeline (Robin et al. 2024) will be used to segment the images (i.e., define the particle contours; see Fig. 19) and then to analyse the size distribution and the detailed shape of particles on the surface of Phobos. This semi-automatic approach to identifying the particle contours removes subjectivity and enables more images to be processed in a shorter amount of time while still including manual verification to ensure particles are correctly identified. The morphological pipeline is fully automatised. Using the particle contour coordinates as an input, the pipeline calculates the size (equivalent diameter), axial ratios (ellipsoidal ratio, bounding box ratio), and morphological characteristics (solidity, compactness, eccentricity, sphericity, roundness,..) for each particle. For the resolution-dependent characteristics (roundness and circularity), a pixel threshold size of >30 pixels is recommended. The smallest particle size that can be analysed in the WheelCam images will increase with increasing image binning (Sect. 3.4). For details about this method refer to Robin et al. (2024).

#### 3D reconstruction of the local topography

**Fig. 20:**
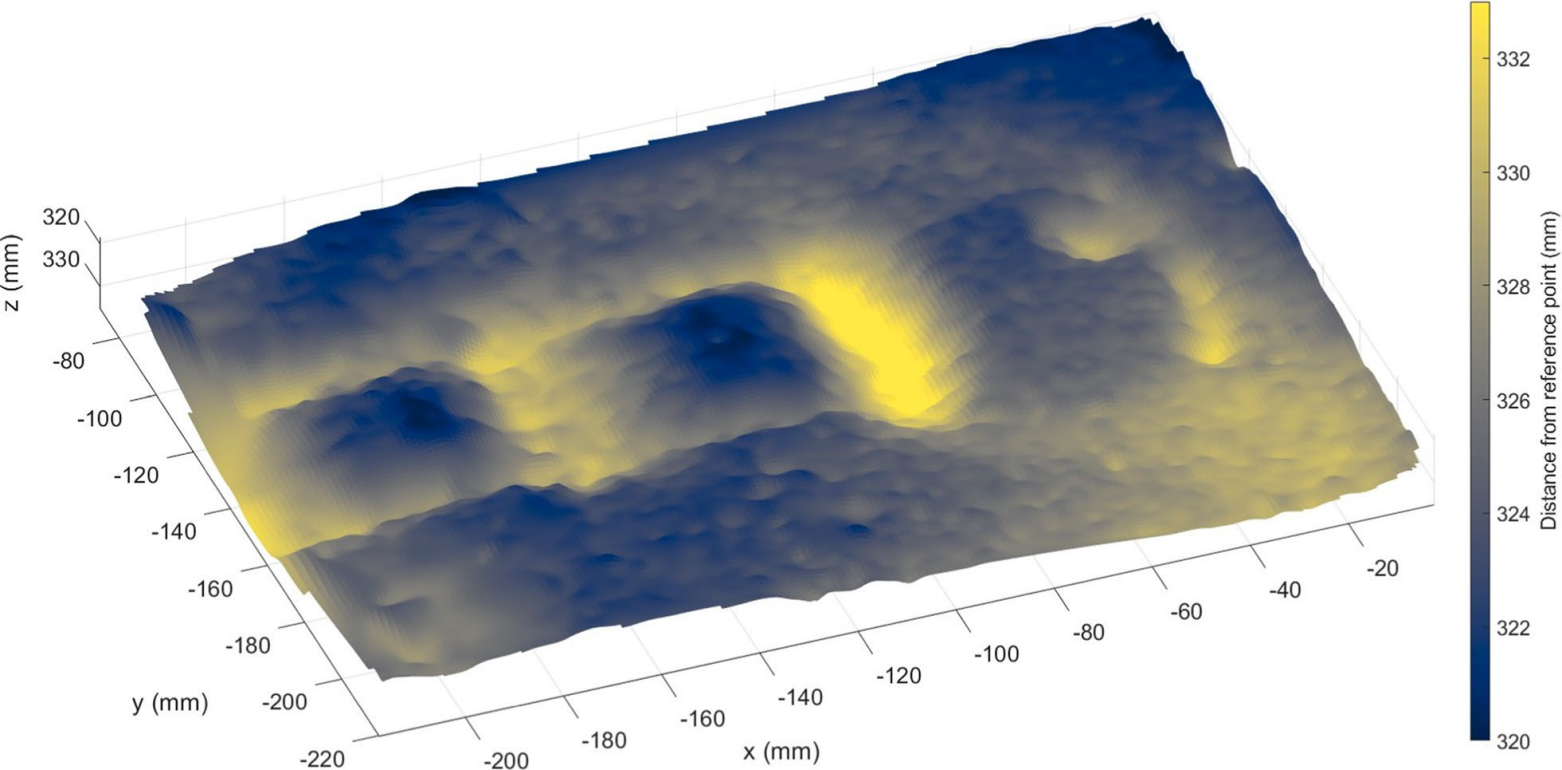
3D reconstruction algorithm. Example 3D model of the reconstructed wheel trench derived from the Structure from Motion method using images acquired with the ISAE-SUPAERO WheelCam testbed. In this example the IDEFIX wheel is rolling on quartz sand. The colour bar indicates the distance (in mm) from a given reference point

A 3D reconstruction algorithm will be used to model the ground topography surrounding the wheel, with a particular interest on modelling the trench left by the IDEFIX wheel. This algorithm is based on the Structure from Motion (SfM) method (Westoby et al. 2012). The process begins with selecting an initial pair of images, and detecting and matching points of interest. By knowing the relative displacement between these two images, the matched points can be triangulated to produce an initial 3D scaled point cloud. In subsequent steps, new points are detected in the following image, and matched with the 3D points, allowing for the estimation of the next camera position. Once this new camera position is determined, further triangulation can be performed and additional 3D points are added into the initial point cloud. A bundle adjustment is then applied to optimize both the 3D points and the camera positions. This iterative process is repeated for every image in the sequence. The result is the complete 3D point cloud with the position of the camera for each image. From this, a surface reconstruction can be computed. An example test case for the algorithm, using the ISAE-SUPAERO WheelCam test bed data, can be seen in Fig. 20. The shape of the trench left by the IDEFIX wheel rolling in quartz sand is clearly visible. In the case of binned images (Sect. 3.4), the resolution of the 3D surface reconstruction will be reduced, but this will not affect the determination of the general trench morphology. For more details about this method refer to Amsili et al. (2025).

#### Rover velocity

**Fig. 21:**
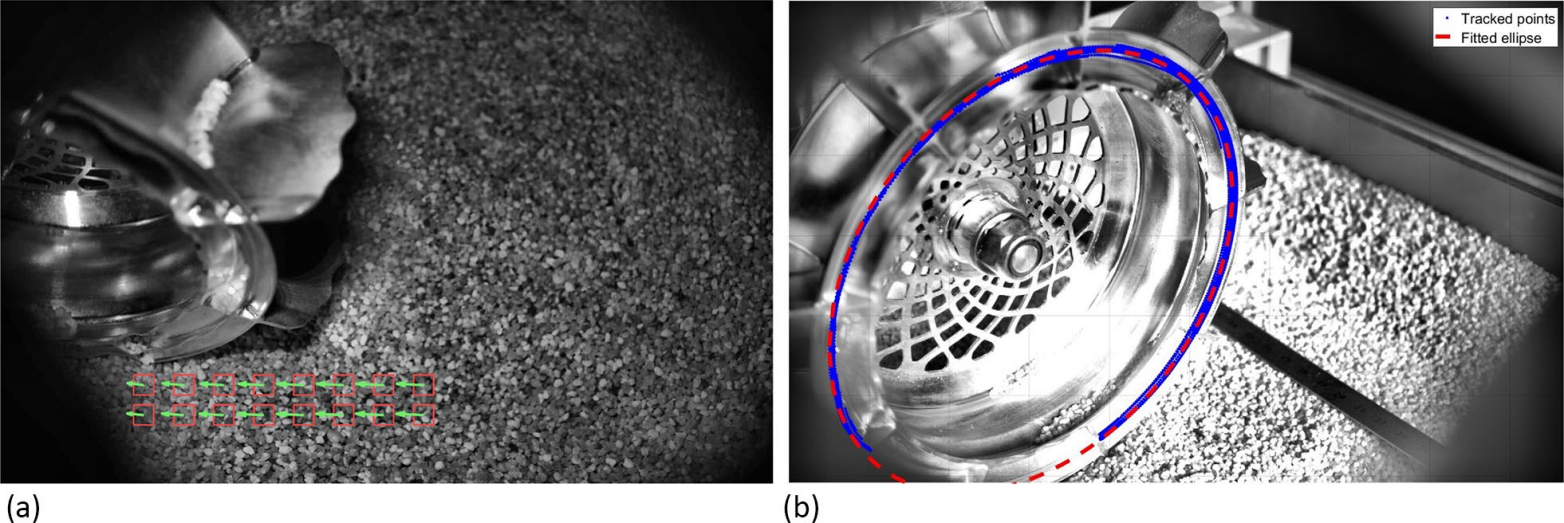
Linear and angular velocity determination. **a** The linear velocity of the rover can be determined using the apparent motion of the regolith particles (green arrows) with respect to the camera. **b** The angular velocity of the wheel can be determined through feature tracking of points on the IDEFIX wheel. The blue points show the tracked features, and the red dashed line is the best fit ellipse, used to calculate the homography matrix before the angular velocity determination

Two methods are considered for computing the linear velocity of the rover from the WheelCam images. The first approach uses the output of the SfM algorithm (see above), which estimates the position of the camera in each frame. By knowing the time at which each image frame was captured, it is possible to calculate the linear velocity of the camera and, therefore, the rover. The second method relies on using an optical flow methodology to analyse the apparent velocity of the ground with respect to the camera. The optical flow algorithm constructs a global velocity vector field of the image set (Fig. 21, left), thus allowing the relative camera—ground movement to be determined from image to image. By computing the translational velocity of the ground (i.e., the regolith particles) with respect to the camera, the rover’s linear velocity can be determined.

By analysing the WheelCam images, the angular velocity of the wheel can also be computed. Feature tracking is applied to points on the wheel’s edge in several images during rotation (Fig. 21, right). An ellipse is then fitted to these points to define the outline of the wheel. By comparing this ellipse to a circle, an homography matrix is estimated. The ellipse-to-circle homography matrix is then used to transform the detected points on the wheel’s edge into a circle. The points tracked along the rover wheel, and the timestamps of the corresponding images, can then be used to calculate the instantaneous angular velocity of the wheel. As long as there are features visible in the images of the wheel, the rover velocity determination is not expected to be significantly affected by any potential binning of the WheelCam images (Sect. 3.4).

## Conclusions

The French–German IDEFIX rover will be deployed to the surface of Phobos late 2028/early 2029 by the JAXA Martian Moons Exploration (MMX) mission. The IDEFIX rover will attempt wheeled-locomotion on a small body surface, in a very low-gravity environment, for the first time.

The WheelCams are two panchromatic cameras placed on the underside of the IDEFIX rover with the front WheelCam observing the left front wheel, and the rear WheelCam observing the front of the left back wheel and also the trench made by the left front wheel. Each WheelCam instrument consists of the detector, the optics and a set of co-located LEDs (white and three different colours: 590, 720 and 880 nm). In order to have the optimal view of the ground, the WheelCams have an unconventional alignment with the optics being tilted by approximately 3$^{\circ }$ with respect to the detector. The WheelCams are protected by a transparent shutter that will be opened after the rover deployment and uprighting sequence is complete. The WheelCams can be operated in both an imaging and a movie mode, the latter is intended to be used during driving.

The WheelCams will provide in-situ images of the surface of Phobos allowing us to examine the mechanical and dynamic properties of Phobos’ regolith by observing the surface and the interactions between the rover wheels and the regolith. Specifically, the WheelCam observations of the IDEFIX wheels and the trench will provide estimates of sinkage, cohesion, friction, and bearing capacity - all key parameters in evaluating whether the surface can support a stable landing or will behave unpredictably (e.g., excessive slippage or collapse). These insights reduce uncertainties, inform engineering expectations, and ultimately mitigate risks associated with relying solely on assumptions. Therefore, in addition to being used to support the interpretation of data obtained by instruments onboard the main MMX spacecraft, and connect the in-situ data to the remote observations, we expect that the WheelCam data will also play an important role in de-risking the MMX sampling operations.

In this paper we have described in detail the WheelCam science objectives including discussing how these objectives will be achieved from the WheelCam images. The technical details of the WheelCam instrument have been provided and the characterisation of the WheelCam flight models, including dark measurements, under-light measurements and the geometrical calibration have been described. Finally, we presented the on-going activities to prepare for the WheelCam operations on Phobos, and the subsequent image processing and interpretation. In particular, we present the soft-sphere Discrete Element Method simulations performed to understand the influence of the low-gravity environment on sinking and driving behaviour and the single-wheel WheelCam testbed developped at ISAE-SUPAERO that recreates the scene that the WheelCams will observe during the IDEFIX mission. The testbed can be used to compare wheel sinkage and driving performance for different types of surface materials but the main objective of the testbed is to develop the image processing tools for the WheelCams. Some example image processing techniques that will be applied to the IDEFIX WheelCam images have been presented briefly and the details will be presented in future work.

## Data Availability

Not applicable.
